# Ubiquitin‐like SUMO protease expansion in rice (*Oryza sativa* L.)

**DOI:** 10.1002/tpg2.70200

**Published:** 2026-02-12

**Authors:** Kawinnat Sue‐ob, Eshan Sharma, Cunjin Zhang, Rahul Bhosale, Ari Sadanandom, Andrew R. Jones

**Affiliations:** ^1^ Institute of Systems, Molecular and Integrative Biology, Department of Biochemistry, Cell and Systems Biology University of Liverpool Liverpool UK; ^2^ Department of Biosciences Durham University Durham UK; ^3^ School of Biosciences University of Nottingham Nottingham UK; ^4^ Department of Botany, Faculty of Science Chulalongkorn University Bangkok Thailand

## Abstract

SUMOylation is a protein post‐translational modification that is essential for plant growth and response to changing environments. However, past work in this area has mainly focused on simple sequence similarity methods for discovering SUMOylation genes, often using orthologue mapping from yeast (*Saccharomyces cerevisiae*) or *Arabidopsis* (*Arabidopsis thaliana*). In this work, we employed a range of computational techniques and approaches to describe and characterize the SUMOylation machinery in Asian rice (*Oryza sativa*), a globally important stable crop, and where the SUMOylation system has been shown to play key roles in responses to biotic and abiotic stresses. We describe and analyze the Ubiquitin‐Like Protease (ULP) system at the phylogenetic, transcriptional, and protein structural levels, with a focus on the rice reference genome, a well‐annotated rice population reference panel (RPRP), and wild rice genomes. Our analysis revealed the expansion of ULPs in the reference genome and RPRP set (32–45 ULPs) compared to wild rice (9–36 ULPs), raising an intriguing hypothesis about the expansion of the ULP family being driven by selective breeding pressure. We provide evidence of potential functional ULPs and their possible roles in biotic and abiotic stress responses in cultivated rice. These insights offer valuable resources for future rice breeding and crop improvement.

## Core Ideas

- Domesticated Asian rice species contain a greater number of ubiquitin‐like SUMO proteases (ULP) than wild relatives of rice.
- Novel ULPs in *Oryza sativa* subspecies *japonica* cultivar Nipponbare are involved in responses to biotic and abiotic stresses.
- We present a study of a ULP gene family in rice at the pan‐genome level.
- We utilized structural biology to identify the functional ULPs.

## Plain Language Summary

Plants require a process called SUMOylation to grow properly and respond to stresses. We used bioinformatic tools to study a group of proteins involved in SUMOylation, known as SUMO proteases (Ubiquitin‐Like Proteases), which help regulate this process in cultivated Asian rice, and compared them with those in wild relatives of rice. We found that these genes have increased in number in cultivated rice, possibly as a result of breeding. These proteins also play important roles in helping rice cope with different types of stress, such as salt and drought stresses. Our findings may contribute to improving rice breeding in the future.

**Abbreviations**
ABA: abscisic acid
BLAST: Basic Local Alignment Search Tool
DeSI: DeSUMOylating Isopeptidase
ELS: ESD4‐Like SUMO Protease
FPKM: fragments per kilobase of transcript per million mapped reads
FUG1: Fourth ULP Gene Class 1
HPY: high ploidy
IRGSP: International Rice Genome Sequencing Project
MSA: multiple sequence alignment
Os_cFUG1: *Os* current FUG1
Os_nFUG1: *Os* novel FUG1
OTS: Overy tolerant to salt
RAP‐DB: Rice Annotation Project database
RGAP: Rice Genome Annotation Project
RPRP: Rice Population Reference Panel
SAE: SUMO activating enzyme
SCE: SUMO conjugating enzyme
SUMO: small ubiquitin‐like modifier
ULP: Ubiquitin‐Like Protease

## 1 INTRODUCTION

SUMOylation is a post‐translational modification found across eukaryotes. The mechanisms of SUMOylation are best understood in model organisms such as *Saccharomyces cerevisiae*, *Arabidopsis thaliana* (Roy & Sadanandom, 2021), and *Homo sapiens* (Celen & Sahin, 2020). The key protein of this mechanism is SUMO (Small Ubiquitin‐like MOdifier), a ∼100 amino acid (aa) protein with a conserved diglycine (‐GG‐) motif near the C‐terminus, followed by a short tail sequence in the premature protein. Pre‐mature SUMO is cleaved at the C‐terminal tail to reveal the di‐Glycine by a ULP (Ubiquitin‐Like Protease). In *A. thaliana*, the mature SUMO is activated by a complex of SAE1a/b and SAE2 (SUMO activating enzymes) in an ATP‐dependent process. The activated SUMO is then conjugated to SCE1 (where SCE is SUMO conjugating enzyme) (E2 conjugating enzyme) and subsequently transferred and ligated to a lysine (K) residue on a target protein by SIZ1 or HPY1 (where HPY is high ploidy) (E3 ligases). The SUMO can also be polymerized by the function of PIAL1/2 (protein inhibitor of activated STAT‐like 1/2). The effects of SUMOylation of a target include localization, complex formation, interaction with other proteins, enzyme activities, or prevention from Ubiquitination (Roy & Sadanandom, 2021). Finally, SUMO proteases such as the DeSUMOylating Isopeptidase (DeSI) and ULP types can cleave the SUMO off the targets, allowing targets and the free SUMO to be recycled.

The SUMOylation system plays various regulatory roles in plant vegetative development and maturation stages at almost every gene regulation step, including chromatin modification/remodeling, epigenetics, transcriptional activation/repression, RNA processing, protein trafficking/localization, and enzyme activities (Augustine & Vierstra, 2018; Benlloch & Lois, 2018; Roy & Sadanandom, 2021). In rice, OsSIZ2, an E3 ligase, has been shown to modulate developmental processes, phosphate (Pei et al., 2017), and nitrogen homeostasis (Pei et al., 2019; Wang et al., 2015) and play a role in reproduction (Pei et al., 2019). Among SUMO proteases in rice, OsFUG1 is involved in regulating development and fertility, OsELS1 contributes to the control of flowering time (Rosa et al., 2018), and OsOTS1 plays a role in seed germination and root development (Srivastava et al., 2016a).

Beyond development, SUMOylation also plays crucial roles in plant response to both abiotic and biotic stresses. Overexpression of OsSCE1 (E2) reduced tolerance to drought stress, and vice versa in OsSCE1‐knocked‐down plants (Nurdiani et al., 2018). OsSCE1‐3 were up‐regulated during drought treatment (Joo et al., 2019). Interestingly, they also responded to abscisic acid (ABA), gibberellin, glucose, and H_2_O_2_ treatments (Joo et al., 2019). The overexpression of OsSIZ1 (E3) in *Arabidopsis* enhances tolerance to several stresses, including drought, heat, and salt (Mishra et al., 2018). Overexpression of OsOTS1 (ULP) in salt stress treatments promotes root growth and results in enhanced salt tolerance (Srivastava et al., 2016a), whereas depletion of OsOTS1/2 reduces salt tolerance but increases drought tolerance (Srivastava et al., 2017). For biotic defense mechanisms, proteomic analyses show that the level of SUMO‐conjugated proteins was increased after pathogen attack, such as *Pseudomonas syringae* (Bailey et al., 2016; Ingole et al., 2021). Given its regulatory potential, the SUMOylation system has been proposed as a potential target for crop improvement (as reviewed in Rosa et al. [2019] and Clark et al. [2022]).

Previous studies have attempted to identify SUMOylation genes in crops such as rice, *Oryza sativa* subspecies *japonica* cultivar Nipponbare, hereafter *O. sativa* spp. *japonica* (Garrido et al., 2018; Orosa et al., 2018; Rosa et al., 2018; Teramura et al., 2021), maize, *Zea mays* (Augustine et al., 2016 *)*, tomato, *Solanum lycopersicum* (Augustine et al., 2016; Zhang et al., 2018), potato, *Solanum tuberosum* (Augustine et al., 2016; Ghimire et al., 2020), and soybean, *Glycine max* (Li et al., 2017), using sequence similarity‐based bioinformatics tools. These studies typically query known SUMOylation protein sequences from *A. thaliana* and *S. cerevisiae* (review in Clark et al., 2022). Among monocots, SUMO components are best characterized in maize and rice (Clark et al., 2022).

In rice, by searching for homologous genes of *AtSUMO1* and *ScSMT3*, studies have identified six *OsSUMO* modifiers (Augustine et al., 2016; Rosa et al., 2018; Teramura et al., 2021). A more recent study claimed a putative identification of *OsSUMO7* (Ibrahim et al., 2021), but this appears to be a misidentification of URM1 (100% sequence identity to Rice Annotation Project Database (RAP‐DB): Os07g0466300, Ubiquitin‐related modifier), involved in Urmylation. Currently, two E1 activating enzymes (OsSAE1 and OsSAE2), three E2 conjugating enzymes (OsSCE1, OsSCE2, and OsSCE3), and three E3 ligases (OsSIZ1, OsSIZ2, and OsHPY2) have been identified in rice, but no E4 ligase gene has been reported (Rosa et al., 2018).

Unlike the other SUMO components, SUMO proteases have highly divergent sequences at the N‐terminus, which limits the discovery using basic local alignment search tool (BLAST)‐based methods. Furthermore, as proteases, the catalytic domains (HxNCN for DeSI and HxDxC for ULPs) are crucial for deSUMOylation (Kurepa et al., 2003; Shin et al., 2012). By searching for ULPs with conserved catalytic domains in the Rice Genome Annotation Project (RGAP) rice database, previous studies have suggested there are 12–22 ULPs in rice (Garrido et al., 2018; Srivastava, et al., 2016b; Yates et al., 2016). However, only 7 OsULPs (OsFUG1, OsELS1, OsSPF1, OsELS2, OsOTS1, OsOTS2, and OsOTS3) have been experimentally validated for the protease function (Rosa et al., 2018; Srivastava et al., 2016b). An expansion of SUMO proteases, relative to *Arabidopsis*, was also observed in maize (Yates et al., 2016). There has been past speculation that expansion of ULPs in crops could have resulted from domestication and could give rise to target specificity during deSUMOylation (Garrido et al., 2018; Morrell & Sadanandom, 2019; Verma et al., 2018). DeSIs, on the other hand, are a relatively recent discovery. They were first characterized in *Arabidopsis* (Orosa et al., 2018; Roy & Sadanandom, 2021; Seok et al., 2021) and more recently in *O. sativa* spp. *japonica* but without molecular functional validation (Lian et al., 2024).

Despite the importance and conservation of SUMOylation in plant systems, there are gaps in our knowledge regarding the SUMO components, and the molecular mechanisms are elusive in crops (Clark et al., 2022). Identifying SUMOylation‐related genes is a crucial first step in studying SUMOylation in crops. In this study, we aimed to profile SUMOylation components in rice (*O. sativa*), as there is an apparent and unusually large expansion of ULP proteases in rice. We also wished to explore variation in the profile of ULPs across the rice pan‐genome, now that there are leveraged newly available multiple high‐quality genome assemblies and gene models available in the so‐called rice population reference panel (RPRP) genome set (Zhou et al., 2023). Furthermore, we performed computational validation using structural biology and assessed public transcriptomic data to provide strong evidence for the functionality of novel ULPs that were predicted.

**Core Ideas**

- Domesticated Asian rice species contain a greater number of ubiquitin‐like SUMO proteases (ULP) than wild relatives of rice.
- Novel ULPs in *Oryza sativa* subspecies *japonica* cultivar Nipponbare are involved in responses to biotic and abiotic stresses.
- We present a study of a ULP gene family in rice at the pan‐genome level.
- We utilized structural biology to identify the functional ULPs.

## 2 METHODS

### 2.1 Identification of ULPs in *O. sativa*

Our first analysis aimed at discovery of ULPs in the reference genome *O. sativa* (International Rice Genome Sequencing Project [IRGSP]). We queried well‐characterized ULPs from *S. cerevisiae* and *A. thaliana* to the two rice genome annotations: RAP‐DB (Sakai et al., 2013) and RGAP (previously called “MSU,” v7, Kawahara et al., 2013) using BLASTP and TBLASTN (blast+/2.9.0; Camacho et al., 2009). The BLAST results were filtered with *e*‐values < 0.05. The significant results from BLAST were analyzed for protein domains by InterProScan v5.66‐98.0 (Jones et al., 2014) with its default parameters.

The sequences bearing the ULP domain (IPR003653), as identified by InterProScan (Jones et al., 2014), were subjected to alignment using MUSCLE v5 (https://hub.docker.com/r/pegi3s/muscle, López‐Fernández et al., 2021). Specifically, sequences displaying alignment with known ULPs from yeast and *Arabidopsis*, particularly within the catalytic domain characterized by the HxDxC motif, were retained as ULP candidates. Proteins derived from the RAP‐DB and RGAP gene models were aligned separately with known ULPs from yeast and *Arabidopsis*.

### 2.2 ULP genes identification in the RPRP and wild relatives of rice

We used BLASTP to search for the ULP orthologues within proteins annotated in the RPRP genomes: Aromatic (*Os*ARC), Indica (*Os*GoSa, *Os*IR64, *Os*KYG, *Os*LaMu, *Os*Lima, *Os*LiXu, *Os*MH63, *Os*Pr106, and *Os*ZS97), *Aus* (*Os*N22 and *Os*NaBo), Tropical *Japonica* or Tropjap (*Os*Azu, *Os*CMeo, and *Os*KeNa), Temperate *Japonica* or Tempjap (IRGSP RefSeq–three annotation sets, RAP‐DB, RGAP, and a new annotation set from the Gramene database, called *Os*Nip), and wild relatives of rice (hereafter wild rice): *Oryza brachyantha*, *Oryza glumipatula*, *Oryza longistaminata*, *Oryza nivara*, *Oryza punctata, Oryza rufipogon*, *Oryza barthii*, African cultivated rice (*Oryza glaberrima*) and closely rice‐related grass *Leersia perrieri* (see the genome information in Table S1) by querying 37 ULP protein sequences from the previous step (both RAP‐DB and RGAP models, Table S2). A stringent filter criterion (*e*‐value < 10^−6^) was applied to BLASTP results. Given that searches were performed within genus level (*Oryza*), we would expect orthologues to have low *e*‐values. Significant (protein) matches resulting from BLASTP were next analyzed with InterProScan. Those with a significant match to the ULP domain (IPR003653) were then aligned with the ULP reference proteins from yeast and *Arabidopsis*.

For the RPRP dataset, with the information of pan‐gene set from the “PanOryza” pan‐gene project (Contreras‐Moreira et al., 2025), we were able to map all the ULP candidates to the pan‐gene matrix and investigate at the “pan‐gene” levels. Here a pan‐gene is cluster of genes that have overlapping coordinates following whole genome alignment, which ideally correspond to alleles of the same gene. If gene models have been incorrectly called in some aligned genomes, then a pan‐gene can contain predicted transcript/protein sequences that differ significantly. Protein sequences derived from each pan‐gene were then independently aligned with yeast and *Arabidopsis* ULP proteins (i.e., one alignment per pan‐gene) using three different multiple sequence alignment (MSA) algorithms—MUSCLE, MAFFT, and CLUSTALOmega (https://hub.docker.com/r/pegi3s/muscle, https://hub.docker.com/r/pegi3s/mafft, and https://hub.docker.com/r/pegi3s/clustalomega, docker images; López‐Fernández et al., 2021) to optimize the identification of conserved ULP active sites. If results from MSAs differed in the aligned residues at the known active sites (from yeast and *Arabidopsis*), we selected if a given protein had the required motifs aligned in at least one set of alignment results, then it was called as a correct match to go forward for the next analysis step. For wild relatives of rice, we aligned all ULP candidate proteins (from BLASTP and InterProScan) with known yeast and *Arabidopsis* ULPs from each wild relative of rice one at a time.

### 2.3 Phylogenetic analysis

A phylogenetic tree was generated, derived from protein MSAs for *S. cerevisiae*, *A. thaliana*, *L. perrieri*, wild relatives of rice, African rice, and RPRP, by the neighbor‐joining method with 1000 bootstraps in MEGA X (Kumar et al., 2018) and visualized/annotated in iTOL (Letunic & Bork, 2024). The 50 sub‐clusters were derived from cutree function in ape library (Paradis & Schliep, 2019). The yeast ULP1 (ScULP1) was used as the outgroup.

### 2.4 Selection of representative genes from the RPRP pan‐genes

To select a representative ULP gene from within the pan‐genes from the RPRP set, we selected a confident ULP candidate (i.e., containing the ULP domain and correct catalytic sites) following the order of genomes/annotations sets within the pan‐gene matrix (Contreras‐Moreira et al., 2025), that is, RAP‐DB, OsNip (Gramene gene model), and RGAP (from the IRGSP Nipponbare reference genome), respectively. If the pan‐gene did not contain a Nipponbare gene member, or those members did not pass the ULP identification criteria, a representative was picked in the following order: *Os*Azu, *Os*CMeo, *Os*Pr106, *Os*KeNa, *Os*ARC, *Os*ZS97, *Os*N22, *Os*MH63, *Os*NaBo, *Os*LiXu, *Os*GoSa, *Os*LaMu, *Os*KYG, *Os*IR64, and *Os*Lima (Contreras‐Moreira et al., 2025).

### 2.5 Protein disorder region prediction

We utilized metapredict v2.61 (Emenecker et al., 2021) to identify disordered regions within protein sequences. The representative ULP sequences from pan‐gene clusters and all ULPs from the eight wide rice varieties were input into metapredict, and the results were visualized using R.

### 2.6 Protein structure analysis of the ULPs

Protein structures for the ULP representatives from the RPRP pan‐genes and all identified ULPs in wild relatives of rice were predicted using ColabFold (https://github.com/YoshitakaMo/localcolabfold; Mirdita et al., 2022). The ColabFold parameters were set as generating three models (–num‐models 3), using amber for the structure refinement (–amber), and running on graphics processing unit (–use‐gpu‐relax). Only the relaxed_rank_001 structures were further investigated. We measured the distances between the three AAs at the catalytic site (angstrom, Å) using an in‐house Python script (get_distancePDB_loop_shortName.py and BioPython version 1.81) and visualized using PyMOL 2.1.1 (Schrodinger, 2018).

### 2.7 RNA‐sequencing analysis

Public rice RNA‐sequencing datasets were collected and re‐analyzed by Yu et al. (2022) using a uniform pipeline. Briefly, the fastq files were mapped to RGAP genome annotation using HISAT2 (Kim et al., 2019). The duplicated reads were removed by using SAMtools (rmdup), and the transcript abundance (Fragments Per Kilobase of transcript per Million mapped reads; FPKM) was calculated using StringTie (Pertea et al., 2015). FPKM values of ULPs (RGAP models) were derived from Public RNA‐seq Database (https://plantrnadb.com/ricerna/), scaled (*z*‐score), clustered by default parameters (complete method and Euclidean distance), and visualized using ComplexHeatmap library in R (Gu et al., 2016).

To gather the expression of ULP genes at single‐cell resolution, RDS files for scRNAseq experiments were retrieved from NCBI GEO GSE232863 (X. Wang et al., 2025) and GSE251706 (Zhu et al., 2025). Seurat (v5.2.1; Hao et al., 2024) was used to calculate the expression levels of ULP genes (RGAP/RAP‐DB annotations) from the RDS object. Log‐normalized pseudo‐bulk values for each identity class were generated using AggregateExpression and plotted as a heatmap using ComplexHeatmap R package (Gu et al., 2016).

## 3 RESULTS

### 3.1 Identification of ULPs in the *O. sativa* reference genome

To identify ULPs in the *O. sativa* IRGSP (Nipponbare) Reference Sequence (hereafter IRGSP RefSeq), we performed a two‐step identification approach. First, homologs of the two yeast ULPs (ScULP1 and ScULP2) and eight *Arabidopsis* ULPs (AtOTS1, AtOTS2, AtELS1, AtELS2, AtEDS4, AtSPF1, AtSPF2, and AtFUG1) were searched (using BLASTP and TBLASTN) against the two IRGSP RefSeq annotation sets (RAP‐DB and RGAP). Significant results (*e*‐value < 0.05) were identified and then analyzed for protein domains using InterProScan. After performing the MSA, we carried forward and retained only those proteins from RAP‐DB or RGAP gene models that had the catalytic domain (HxDxC) when aligned with the ten known ULPs from yeast and *Arabidopsis* (Figure S1). The alignment at the conserved positions of the catalytic motif (HxDxC) distinguishes ULPs from other proteases in the deubiquitination family, such as UBPs (ubiquitination) (Figure S2). Our analysis identified 33 ULPs (RAP‐DB) or 37 (RGAP) and 37 non‐redundant models in IRGSP RefSeq that contain the catalytic domains (Table S2). This indicates the inconsistency between the two gene models and highlights the importance of cross‐referencing the genes of interest in both models, which should always be considered.

**FIGURE 1:**
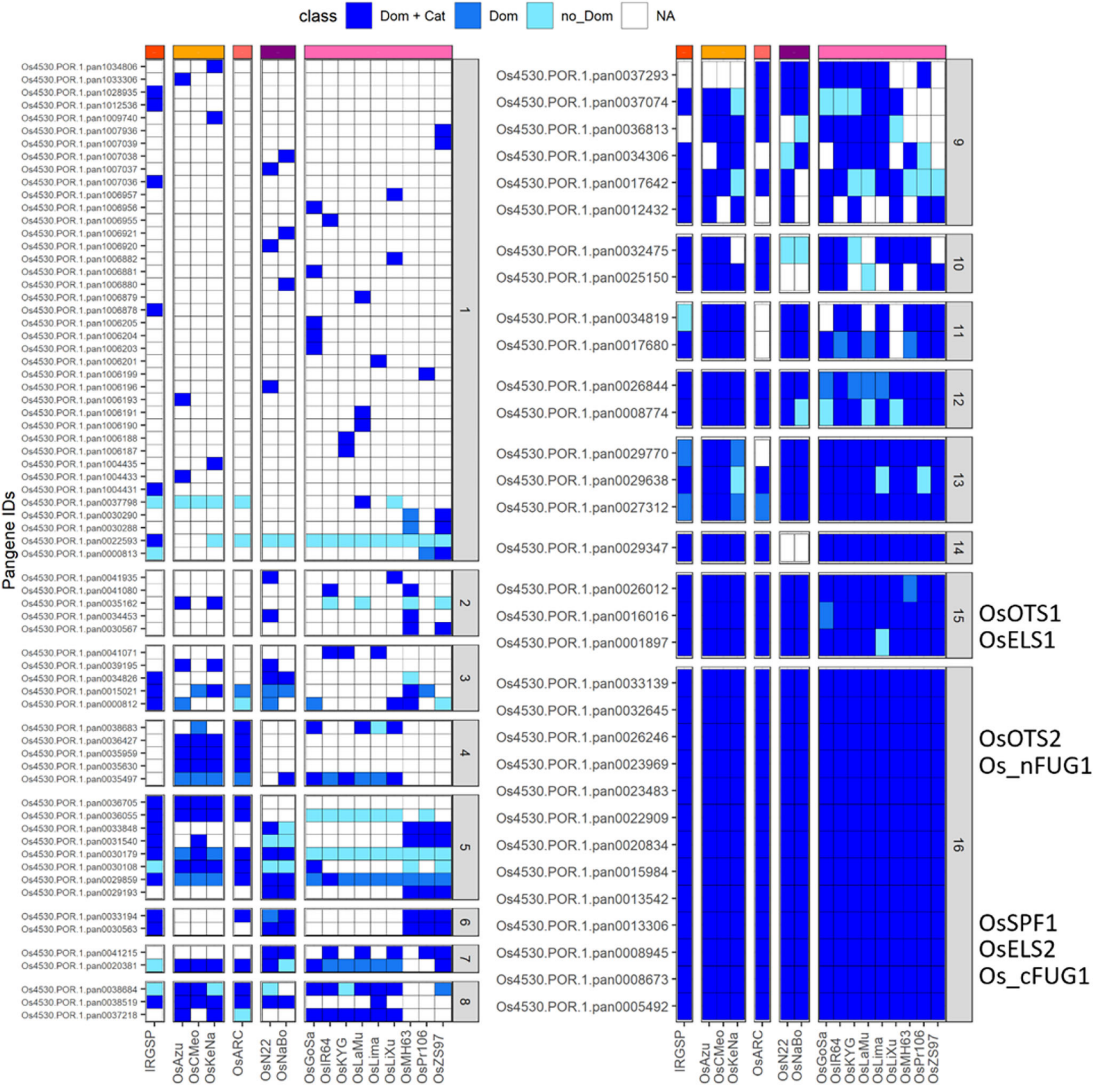
Occupancy of ubiquitin‐like protease (ULP)‐domain in rice population reference panel (RPRP) genomes. 101 Pan‐gene ids of ULP genes are shown in rows, and the groups of RPRP rice are separated by columns (Tempjap, Tropjap, Aro, Aus, and Indica, respectively). The pan‐gene clusters are separated by the presence of ULP domains among 16 populations (domain occupancy). Colors show classification of the genes, ranging from the darkest blue to white if there are ULP domains and correct catalytic sites (Dom + Cat), only ULP domains (Dom), no ULP domains (no_Dom), and no genes (NA) in the pan‐gene cluster.

Secondly, the 37 candidates from the genes identified in the previous step (Table S2) were searched against a recently released additional gene model annotation set for IRGSP, recently produced and released by the Gramene database team (gene id starts with OsNip) using a similar approach, mapped to the pan‐gene matrix, and investigated at the pan‐gene level (see Methods). Next, we further validated the candidates by predicting their 3D structures of the ULP proteins using ColabFold (Mirdita et al., 2022). Finally, the proteins that have conserved catalytic sites with yeast and *Arabidopsis* were finalized as ULPs in IRGSP RefSeq (Table 1; Figure 1).

**TABLE 1:** Summary of ubiquitin‐like proteases (ULPs) in *Oryza sativa* spp. japonica.

Pan‐id	RAP‐DB	Gramene	RGAP (MSU)	Gene name	Count domain	Count rice
pan0005492	*Os02g0472200*	None	*LOC_Os02g27280*	*OsULP40a*	16	16
pan0008673	*Os03g0344300*	*OsNip_03G017390*	*LOC_Os03g22400*	(*OsFUG1*) *OsELS3*	16	16
pan0008945	Os03g0410100	*OsNip_03G021330*	LOC_Os03g29630	*OsELS2*	16	16
pan0013306	*Os05g0207900*	*OsNip_05G007210*	*LOC_Os05g11770*	*OsSPF1*	16	16
pan0013542	*Os05g0309000*	*OsNip_05G011330*	None	*OsULP40b*	16	16
pan0015984	*Os06g0474600*	*OsNip_06G015830*	*LOC_Os06g28030*	*OsULP40c*	16	16
pan0020834	Os09g0258700	*OsNip_09G002660*	LOC_Os09g08440	*OsULP50a*	16	16
pan0032645	None	*OsNip_09G004490*	*LOC_Os09g12480*	*OsULP45a*	16	16
pan0033139	Os10g0157000	*OsNip_10G003120*	*LOC_Os10g06910*	*OsULP42a*	16	16
pan0022909	*Os10g0474000*	*OsNip_10G014370*	*LOC_Os10g33450*	*OsULP35*	16	16
pan0023483	*Os11g0102700*	OsNip_11G000130	*LOC_Os11g01180*	*OsULP37a*	16	16
pan0023969	*Os11g0214001*	*OsNip_11G007290*	*LOC_Os11g10780*	*OsFUG1*	16	16
pan0026246	*Os12g0606900*	*OsNip_12G021260*	*LOC_Os12g41380*	*OsOTS2*	16	16
pan0001897	*Os01g0355900*	*OsNip_01G016190*	*LOC_Os01g25370*	*OsELS1*	15	16
pan0016016	*Os06g0487900*	*OsNip_06G016450*	*LOC_Os06g29310*	*OsOTS1*	15	16
pan0026012	*Os12g0551900*	OsNip_12G017880	*LOC_Os12g36580*	*OsULP40d*	15	16
pan0029347	Os04g0354300	*OsNip_04G010250*	*LOC_Os04g28590*	*OsULP43a*	14	14
pan0029638	Os04g0583400	*OsNip_04G025050*	LOC_Os04g49394	*OsULP50b*	13	16
pan0026844	None	*OsNip_01G019120*	*LOC_Os01g33520*	*OsULP32*	12	16
pan0008774	Os03g0365250	*OsNip_03G018740*	LOC_Os03g24960 LOC_Os03g24980 LOC_Os03g25000	*OsULP50c*	12	16
pan0017680	Os07g0283600	*OsNip_07G010740*	*LOC_Os07g18300*	*OsULP38*	11	14
pan0032475	None	*OsNip_09G000230*	None	*OsULP46*	10	13
pan0025150	*Os12g0127600*	OsNip_12G001960	None	*OsULP37b*	10	11
pan0012432	*Os04g0639700*	*OsNip_04G028960*	*LOC_Os04g54670*	*OsULP44a*	9	9
pan0037074	None	*OsNip_07G008460*	LOC_Os07g12990	*OsULP50d*	9	13
pan0017642	*Os07g0267400*	*OsNip_07G010020*	None	*OsULP40e*	9	15
pan0034306	*Os11g0646232*	*OsNip_11G022650*	*LOC_Os11g42610*	*OsULP40f*	9	11
pan0038519	*Os12g010358 0*	OsNip_12G000230	*LOC_Os12g0129 0*	*OsULP36*	8	8
pan0033194	Os04g0377400	*OsNip_04G011280*	*LOC_Os04g30860*	*OsULP50e*	6	7
pan0030563	None	*OsNip_06G009810*	*LOC_Os06g13750*	*OsULP48*	6	6
pan0036055	*Os03g0614100*	*OsNip_03G026210*	*LOC_Os03g41780*	*OsULP42b*	5	12
pan0029859	None	*OsNip_05G006220*	*LOC_Os05g10270*	*OsULP50f*	5	16
pan0030179	*Os05g0417800*	*OsNip_05G017400*	*LOC_Os05g34520*	*OsULP45b*	5	16
pan0036705	*Os05g0448000*	*OsNip_05G019120*	*LOC_Os05g37550*	*OsULP43b*	5	5
pan0031540	Os07g0504300	*OsNip_07G016450*	LOC_Os07g32090	*OsULP43c*	5	7
pan0033848	None	*OsNip_11G008380*	*LOC_Os11g12500*	*OsULP50g*	5	6
pan0000812	*Os01g0113000*	*OsNip_01G001010*	*LOC_Os01g02270*	*OsULP44b*	3	8
pan0015021	*Os06g0122600*	*OsNip_06G001600*	*LOC_Os06g03180*	*OsULP44c*	3	8
pan0034826	None	*OsNip_12G012470*	*LOC_Os12g24880*	*OsULP19*	3	4
pan1012536	*Os02g0262500*	None	*LOC_Os02g16240*	*OsULP28*	1	1
pan1007036	None	*OsNip_03G004800*	None	*OsULP30*	1	1
pan1006878	Os03g0365200	None	*LOC_Os03g24990*	*OsULP50h*	1	1
pan1004431	Os08g0429600	*OsNip_08G017070*	*LOC_Os08g33280*	*OsULP50i*	1	1
pan0022593	*Os10g0388600*	OsNip_10G009270	*LOC_Os10g24954*	*OsULP43d*	1	14
pan1028935	*Os11g0235825*	OsNip_11G022640	*LOC_Os11g12780*	*OsULP39*	1	1

*Note*: The pan‐gene identifiers (Os4530.POR.1.panXXXXXXX) of ULPs in International Rice Genome Sequencing Project (IRGSP) RefSeq (Nipponbare_merged) are arranged by the number of domain occupancy (count‐domain) and genome occupancy (count‐rice) in the rice population reference panel (RPRP) set. Gene models containing a ULP domain, as identified by InterProScan, are italicized; those with HxDxC catalytic residues, identified by multiple sequence alignment (MSA), are underlined; and the representative models that were protein‐structured (AlphaFold) are bold. The gene names were assigned based on the hierarchical clusters in Figure 2 and followed by letters according to domain occupancy.

Abbreviations: RAP‐DB, Rice Annotation Project Database; RGAP, Rice Genome Annotation Project.

In total, we identified 45 ULPs in IRGSP RefSeq (Table 1; Figure 1). In this final ULPs list, 12 genes from the initial set (37 genes, RAP‐DB/RGAP) were removed because six protein sequences were unaligned with known catalytic sites (when aligned within the pan‐gene cluster) and six sequences showed incorrect protein structures at catalytic sites, as predicted by ColabFold (see Table S2). In addition, an extra 20 genes were incorporated, indicating these proteins as their sequences were aligned better within their pan‐gene cluster. Our approaches have successfully identified all previously known ULPs in rice, including OsOTS1 (Os06g0487900), OsOTS2 (Os12g0606900), OsFUG1 (Os03g22440), OsELS1 (Os01g25370), OsSPF1 (Os05g11770), and OsELS2 (Os03g29630), as previously described (Rosa et al., 2018). However, OsOTS3 (Os01g53630), which has no aligned catalytic sites after MSA was discarded from the full gene final set. There are 13 ULPs that were found to contain the conserved ULP domains and correctly positioned catalytic sites across all 16 genomes examined (count‐domain) (Table 1; Figure 1).

### 3.2 ULPs are expanded in the RPRP pan‐genome

To further explore the expansion of ULPs in the rice pan‐proteome (derived from the *RPRP* pan‐genome), we identified ULPs from each RPRP genome/proteome using BLASTP, followed by protein domain identification (InterProScan) and MSA. As the identification of ULP relies on the MSA of the catalytic sites, there is, however, uncertainty of MSA that different algorithms produce different results (Blackburne & Whelan, 2013). To minimize these effects, we used MSA by using three different alignment algorithms to align for the ULPs (see method). Then, the identified ULP candidates were mapped to the pan‐gene matrix. The pan‐gene matrix was created via the GET_PANGENES pipelines (Contreras‐Moreira et al., 2023), using the RPRP genomes and gene models as input, to identify the orthologous gene (pan‐gene) in each genome. In total, the ULPs can be mapped to 101 pan‐gene identifiers (after protein structure correction), with “genome occupancy” (Figure S3) and “domain occupancy” (Figure 1) ranging from 1 to 16 across the rice species. The ULPs have an unusual pattern of evolution—with strong enrichment in genes found in only a single genome. The analysis in Contreras‐Moreira et al. (2025) described an approach for classifying protein function according to presence of a given domain across 16 genomes, with those protein domains with mean occupancy < 10 (out of 16) being classed as “highly variable” domains—which are enriched for functions related to disease resistance (among others), and expansion of the gene family appears to be driven by selection pressure. Based on the analysis here, the mean occupancy for the ULP is 5.94, raising the possibility that ULPs have also been under selection pressure.

Among the pan‐gene clusters that have 16 genome occupancies across all 16 rice species (27 clusters, Figure S3), some of them were not identified as ULPs due to the absence of ULP domains or catalytic sites, which may be the result of true differences in proteins across cultivars or faulty gene models in some cultivars. The 16‐domain occupancy group consists of 13 pan‐gene clusters, including known rice ULPs: OsOTS2, OsSPF1, OsELS2, and *Os* current FUG1 (Os_cFUG1) (Figure 1). Two additional known rice ULPs, OsOTS1 and OsELS1, are grouped within the 15‐domain occupancy cluster. This is because OsLima_OTS1 contains a ULP domain detected by InterProScan, but its catalytic sites do not align with the ULPs from yeast and *Arabidopsis*, while OsGoSa_ELS1 lacks the ULP domain (Figure S4).

### 3.3 ULP expansion may be associated with selected breeding in Asian rice

To examine whether the ULP expansion occurs only in cultivated rice species, we applied the same approach (BLASTP, followed by protein domain identification by InterProScan, 3‐MSA, and protein structure modeling) to identify ULP‐encoding genes in eight wild relatives and a cultivated African rice and compared the results with the RPRP pan‐genome.

**FIGURE 2:**
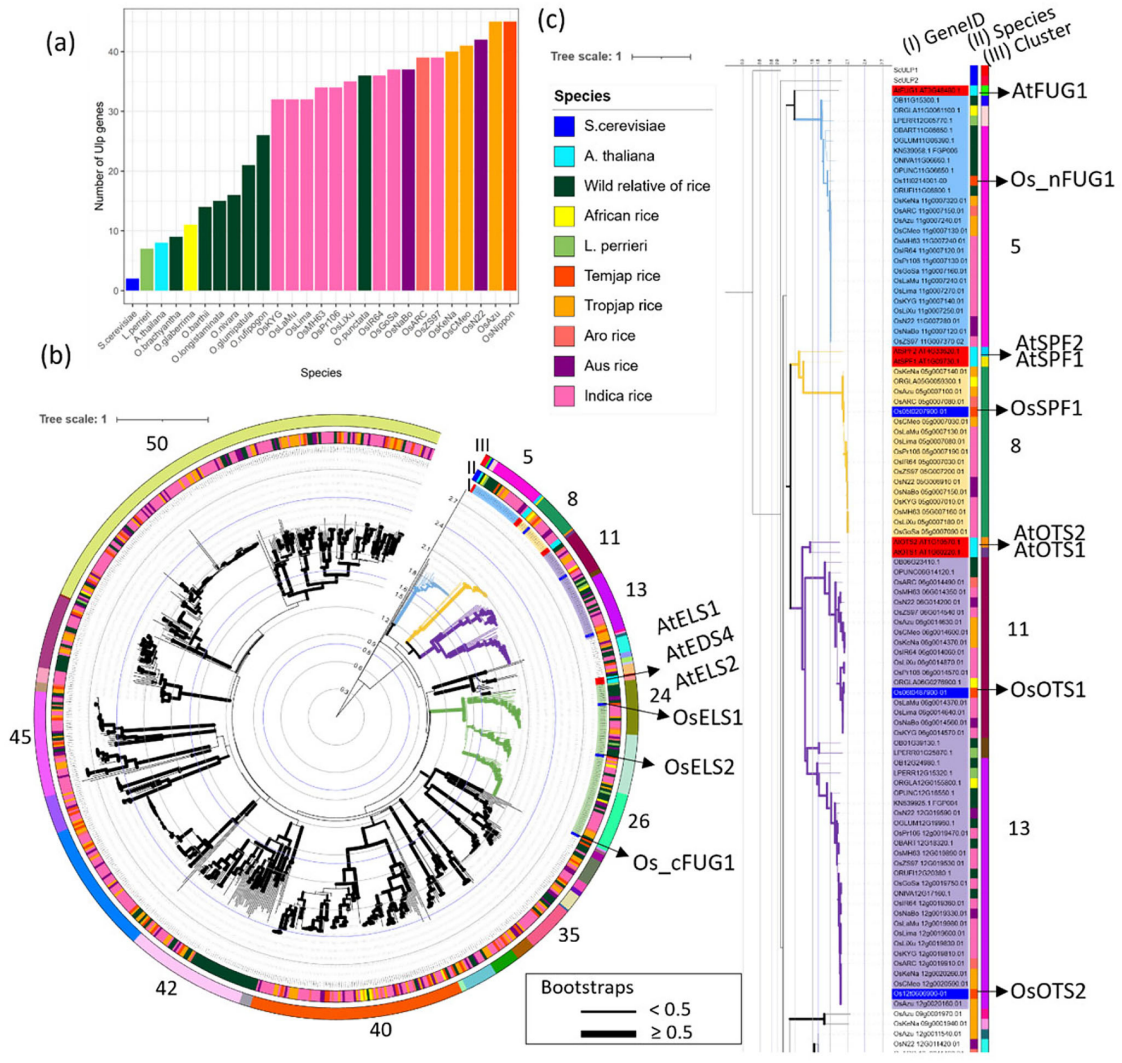
Expansion of ubiquitin‐like protease (ULP) in rice. (a) Count of ULP encoding genes (ranked by count) in yeast, *Arabidopsis*, cutgrass (*Leersia perrieri*), wild rice (*Oryza brachyantha*, *Oryza glumipatula*, *Oryza longistaminata*, *Oryza nivara*, *Oryza punctata, Oryza rufipogon*), African rice (*Oryza barthii*, *Oryza glaberrima*) and *Oryza sativa* RPRP genome set: Aro (*Os*ARC), *Indica* (*Os*GoSa, *Os*IR64, *Os*KYG, *Os*LaMu, *Os*Lima, *Os*LiXu, *Os*MH63, *Os*Pr106, and *Os*ZS97), *Aus* (*Os*N22, *Os*NaBo), Tropical Japonica or Tropjap (*Os*Azu, *Os*CMeo, and *Os*KeNa), Temperate Japonica or Tempjap (ISRGP RefSeq). (b) ULP tree of all species. The tree was constructed by Neighbor Joining (NJ) with 1000 bootstraps. Annotation strips show (I) gene ids, (II) species, (III) subclusters as also represent by numbers. See the order of ULPs of the phylogenetic tree in Table S3. (c) Sub‐cluster of ULPs (clusters 1–15), showing a potential novel OsFUG1 (Os_nFUG1) and the known ULPs in rice: OsOTS1, OsOTS2. The known ULPs in *Arabidopsis* and rice are highlighted in red and blue, respectively. The bootstrapping values over or equal to 50% are shown in thicker clades. The tree was rooted by using yeast ULP1a as an outgroup.

The number of ULPs in wild relatives of rice ranges from 9 to 26, except for *O. punctata*, which contains 36 genes (Figure 2a), while *O. glaberrima*, a cultivated African rice, has 11 ULPs (Figure 2a). We found that the counts of ULPs in the RPRP genomes were higher than those in the African rice and in almost all wild rice, with the exception of *O. punctata* (Figure 2a). Next, we constructed the phylogenetic tree (using neighbor‐joining method with 1000 bootstraps) to show the relationship among known ULPs in yeast, *Arabidopsis*, and newly identified ULPs in cutgrass, wild, African, and domesticated rice (RPRP set) (Figure 2b). The dendrogram was then divided into 50 clusters of ULPs (Table S3). There are clusters containing known rice ULPs: OsOTS1 (Os06g0487900 or LOC_Os06g29310; cluster 11), OsOTS2 (Os12g0606900 or LOC_Os12g41380; cluster 13), and OsSPF1 (Os05g0207900 or LOC_Os05g11770; cluster 8). Additionally, OsELS1 (Os01g0355900 or LOC_OS01G25370), OsELS2 (Os03g0410100 or LOC_Os03g29630), and the currently designated OsFUG1 (Os03g0344300 or LOC_Os03g22400; Os_cFUG1) were assigned to clusters 24–26, respectively (Figure 2b).

Interestingly, we identified a novel rice ULP group within cluster 5 (Figure 2b,c), which is grouped closely related to *A. thaliana* FUG1 (Fourth ULP Gene Class 1). The role of AtFUG1 has been recently revealed as being involved in epigenetic gene silencing (Sureshkumar et al., 2024). In rice, OsFUG1 was discovered by a homology‐based search against RGAP reference genome (Kawahara et al., 2013) and was named OsFUG1 based on a phylogenetic relationship with AtFUG1 (Rosa et al., 2018). However, our analysis suggests that the current OsFUG1 (here called Os_cFUG1) is not a close homologue of AtFUG1, as Os_cFUG1 sits within cluster 26. Within cluster 5, which contains AtFUG1, we see that identified Os11g0214001 is a close homologue, thus making it a more appropriate candidate for renaming as OsFUG1—here designated *Os* novel FUG1 (Os_nFUG1). Cluster 26, which includes Os_cFUG1, also contains ESD4‐Like SUMO Protease (ELS)‐type ULPs. ELS ULPs were initially characterized based on molecular and phenotypic approaches and have been implicated in flowering (Rosa et al., 2018). The close homology between Os_cFUG1 and OsELS1/OsELS2 suggests that Os_cFUG1 should be renamed to OsELS3. Moreover, MSA shows that AtFUG1 is more similar to Os_nFUG1 than Os_cFUG1 (Figure S5). Additionally, there are ULPs in clusters 27–50 that contain ULPs with no closely homologous genes in yeast and or *Arabidopsis*, suggesting that they could be novel groups of ULPs in rice (Figure 2b).

Asian rice species were domesticated from two wild rice species, *O. rufipogon* and *O. nivara* (Jing et al., 2023). Among rice ULPs, *Os_cFUG1* (*Os03g0344300* or *LOC_Os03g22400*) has been identified as one of the domesticated genes that originated from *O. nivara* (Jing et al., 2023). However, our phylogenetic analysis suggests that OsFUG1 (Os_cFUG1) forms a sister clade with ORUFI03G17620 and is more distantly related to ONIVA03G18050, indicating that Os_cFUG1 is likely derived from *O. rufipogon* (cluster 26, Figure 2; Figure S6a). The MSA analysis of Os_cFUG1, ONIVA03G18050, and ORUFI03G17620 reveals two mismatches between Os_cFUG1 and ONIVA03G18050 (99.5% sequence identity), while Os_cFUG1 and ORUFI03G17620 are 100% identical (Figure S6b). AtOTS1 and AtOTS2 are placed into clusters 9 and 10 and have no direct orthologous mapping to OsOTS1 and OsOTS2, in clusters 11 and 13 (Figure 2b,c), indicating the independent duplication of Overy tolerant to salts (OTSs) in *Arabidopsis* and rice. OsOTS1 and OsOTS2 are in sister clades and share a common evolutionary origin. Within each OsOTS clade (clusters 11 and 13, Figure 2b,c), there are orthologous ULP from RPRP rice (varieties of *O. sativa*), as well as other *Oryza* species, suggesting that the divergence of OTS1 and OTS2 occurred before speciation events within the *Oryza* genus. The divergence of OTS1 and OTS2 in monocot species probably pre‐dated the *Oryza* genus, as there are orthologues of OTS2 in the nearest outgroup of the genus *Oryza*: cutgrass (LPERR01G25870.1; cluster 12 and LPERR12G15320.1; cluster 13, Figure 2b,c), although a further exploration of the evolution of OTS genes is beyond the scope of this work.

### 3.4 Sequence and structural analyses of ULPs in wild and domesticated rice

**FIGURE 3:**
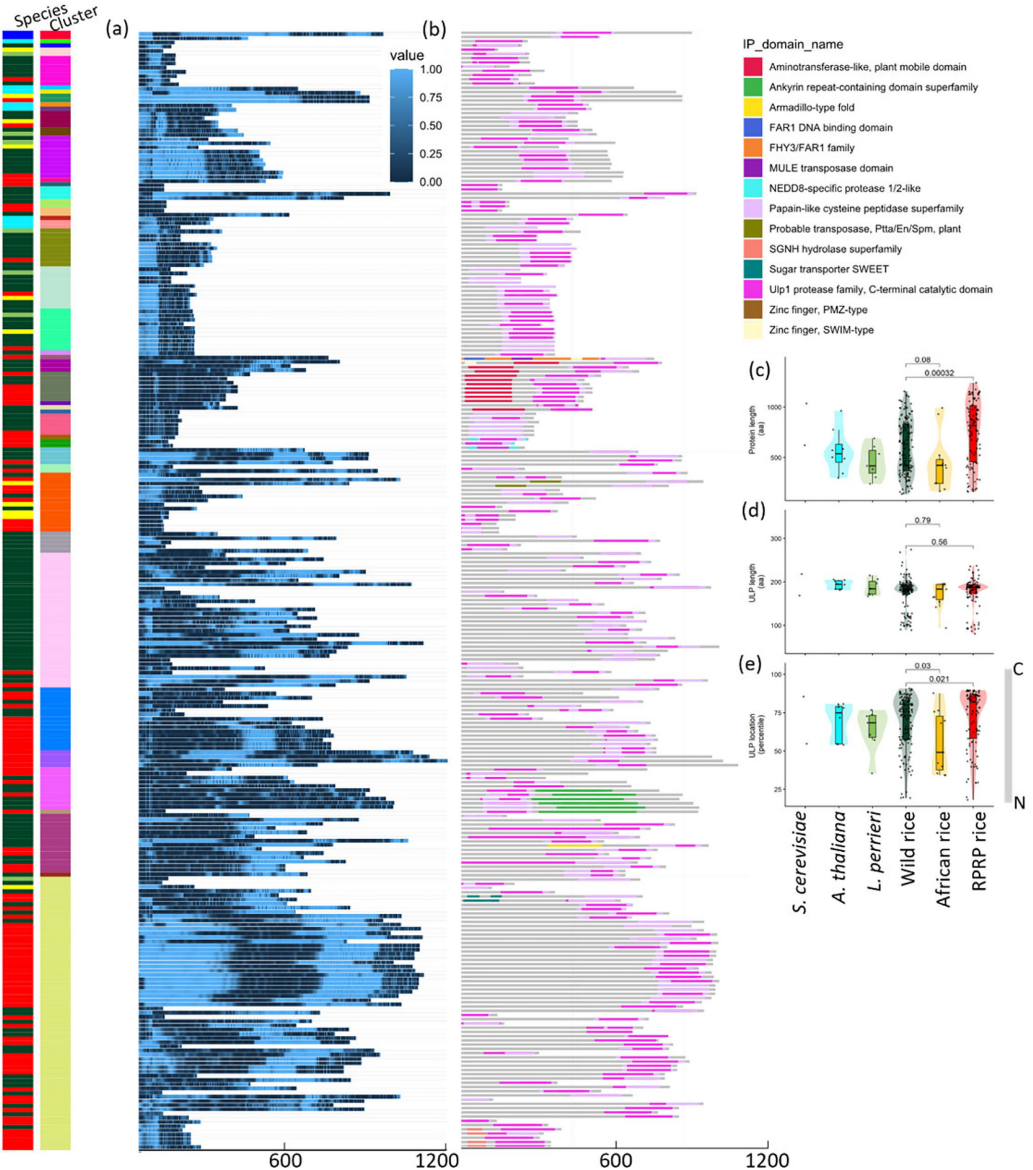
Ubiquitin‐like protease (ULP) protein domains architecture. (a) Disorder regions prediction by metapredict. Scores are ranging from 0 to 1 (light blue) which indicates disorder regions. (b) Protein domains identified by InterProScan. The ULP proteins are ordered by the gene tree in Figure 3. (c) The length (amino acids) of ULPs. (d) The length of ULP domains (amino acids). (e) the ULP domains position on the ULP proteins. Colors in species annotation bar and subparts (c–e) represent species; *Saccharomyces cerevisiae*, *Arabidopsis thaliana, Leersia perrieri*, wild rice, African rice, and rice population reference panel (RPRP) rice (which includes Temperate Japonica; Temjap, Tropical Japonica;Tropjap, Aro rice, Aus rice, Indica rice). Wilcoxon's statistical test was applied for comparing two groups (non‐parametric) and *p*‐values are indicated.

The catalytic domains of ULPs have crucial catalytic sites (HxDxC), which are important for deSUMOylation (Kurepa et al., 2003). We identified the protein domains in the representative ULP candidates (identified from pan‐genes) using InterProScan and assessed the predicted disordered regions via metapredict (Emenecker et al., 2021). Disordered regions are indicated by lighter blue shades (values close to 1), and the more ordered regions are shown in darker blue (Figure 3a). The ULP catalytic domains are typically located closer to the C‐termini of the proteins (Figure 3b). There are overlapping homologous superfamilies of other domains, such as papain‐like cysteine peptidase superfamily (IPR038765), which is shown in light purple (Figure 3b). These ordered regions align with the ULP1 protein domains as suggested by InterProScan, suggesting that there are potential functional domains in these ULPs at the C‐termini as expected. Interestingly, ULPs fused with other domains, such as aminotransferase‐like plant mobile, ankyrin repeat‐containing, and MULE transposase domains, were found in several clusters where there were no orthologue genes in *Arabidopsis*, suggesting independent gene expansion and potential gene sub‐functionalization in rice genus *Oryza* (Figure S7).

The length of candidate ULPs varies across the species. RPRP genomes have significantly (Wilcoxon's test, *p* = 3.2 × 10^−4^) longer ULP protein sequences (Figure 3c), also indicating larger protein structures than wild rice. The ULPs of African rice have similar protein length of ULPs to the wild rice species (Wilcoxon's test, *p* = 0.08). The lengths of the ULP domains are similar across all species and varieties (Figure 3d); however, the positions of the ULP domains within the proteins vary in African rice (Wilcoxon's test, *p* = 0.03) and RPRP rice (Wilcoxon's test, *p* = 0.021) compared to the wild relatives of rice (Figure 3e). In yeast, there are two types of ULPs. The ScULP1 and ScULP2 have their domains located at C‐terminus and in the middle of the protein, respectively. This feature seems to be conserved in *Arabidopsis* and rice, as there are two populations of ULP locations on proteins (Figure 3e). In rice, however, the majority of ULPs have their domains located at the C‐terminus (Figure 3b,e). The protein domain composition and disorder region predictions for all ULPs are presented in Figure S8. We also display the chromosomal locations of all ULPs within the RPRP set in Figure S9, demonstrating that most ULPs are consistently located on chromosomes across genomes, with some exceptions.

**FIGURE 4:**
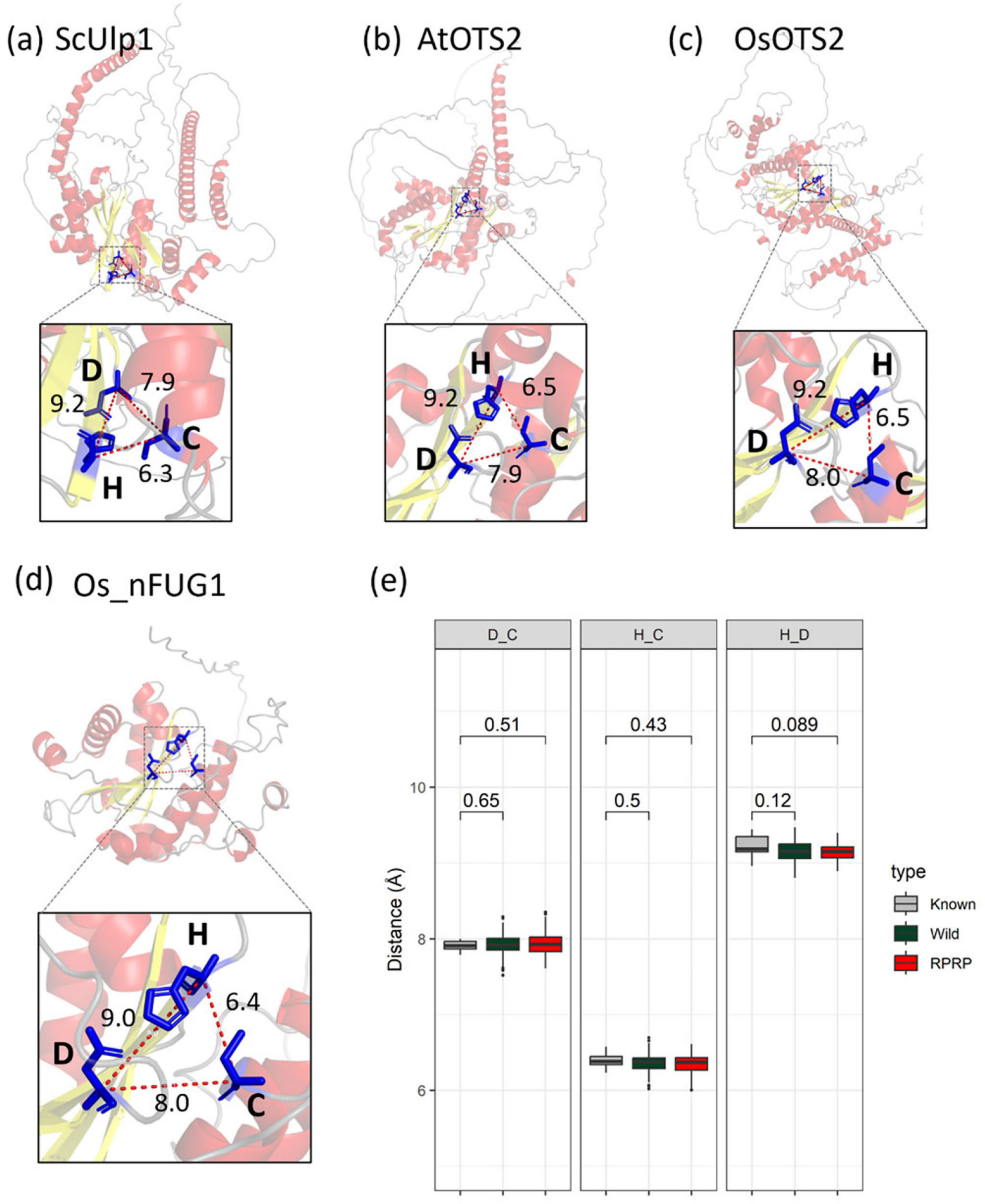
Protein structures predicted by ColabFold of (a) ScULP1, (b) AtOTS2, (c) OsOTS2 and (d) *Os* novel FUG1 (Os_nFUG1). (e) The lengths (angstrom, Å) between the three catalytic sites (C; Cysteine, D; Aspartic acid, and H; Histidine amino acids) of known ubiquitin‐like proteases (ULPs) (ScULP1, ScULP2, AtOTS1, AtOTS2, AtELS1, AtELS2, AtEDS4, AtFUG1, AtSPF1, AtSPF2, OsOTS1, and OsOTS2). Sc: *Saccharomyces cerevisiae*, At: *Arabidopsis thaliana*, Os: *Oryza sativa* with novel ULPs in domesticated (rice population reference panel [RPRP]) and wild relative (Wild) rice. Statistical analysis was done with non‐parametric Wilcoxson's test and *p*‐values are shown.

To assess whether the identified ULPs could potentially function as SUMO proteases, we used AlphaFold2 to predict the structures of the representative RPRP ULP proteins and all ULPs in wild rice. We measured the distances between the three residues that form the ULP catalytic site and compared them to the known ULP catalytic distances from ScULP1, ScULP2, AtOTS1, AtOTS2, AtELS1, AtELS2, AtEDS4, AtFUG1, AtSPF1, AtSPF2, OsOTS1, and OsOTS2. Protein structures of RPRP ULPs that exhibited incorrect catalytic site conformations at the catalytic site, potentially impairing enzymatic activity or substrate binding, were excluded from the list (Figure S3). The catalytic sites of ScULP1, AtOTS2, OsOTS2, and Os_nFUG1 are shown in Figure 4a–d. The average catalytic distances (angstrom, Å) between H and D is 9.22, H and C is 6.39, and D and C is 7.91 (Figure 4e; Table S4). The catalytic site distances in the pan‐gene representatives (RPRP) and wild rice (Wild) were similar and not significantly different from the known ULP reference distances (Figure 4e), suggesting likely SUMO protease activity. However, the D to C distances showed slight variations from the references, suggesting some possible differences between wild and domesticated rice (Figure 4e).

### 3.5 Transcriptomics evidence of novel ULPs in *O. sativa* spp. *japonica*

**FIGURE 5:**
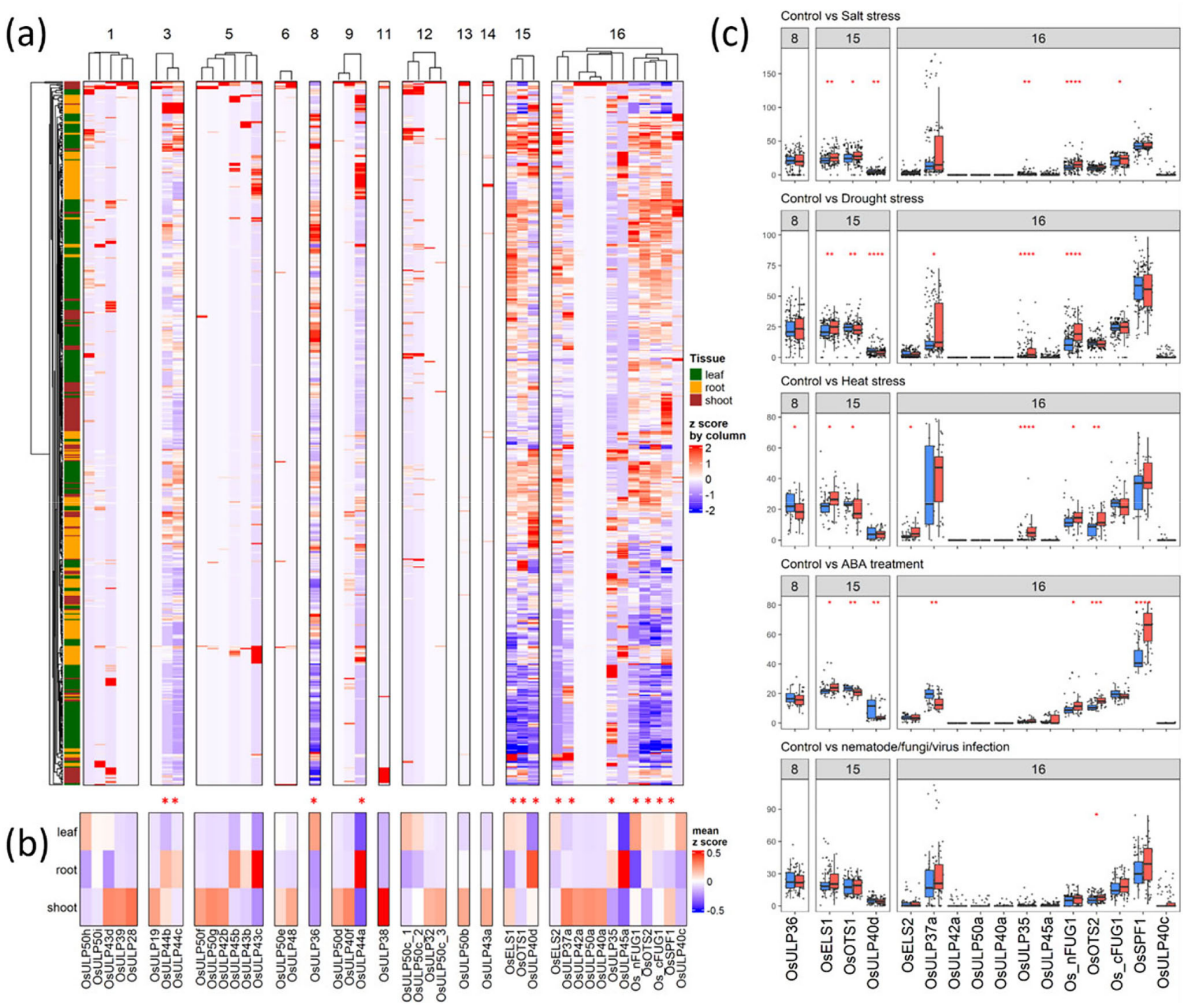
Transcriptomic evidence of the novel ubiquitin‐like proteases (ULPs) in *Oryza sativa* spp. *japonica*. (a) A total 631 publicly available RNA‐seq dataset of *Oryza sativa* leaves (315), shoots (116), and roots (200) showing tissue‐specific patterns of the novel ULPs. The Fragments Per Kilobase of transcript per Million mapped reads (FPKM) values were scaled by genes (*z*‐score by column) then clustered by tissues (row) and within the ULP domain occupancy groups (1–16). (b) Mean of *z*‐score values of ULP expressions by tissues. The ULP genes that were expressed (FPKM > 0) in over 50% of the samples are indicated by asterisks (*). (c) The FPKM values of the 16 ULPs under control (blue) and 5 stress treatments (red): salt (148), drought (188), heat (54), nematode/fungi/virus infection (105), and abscisic acid (ABA) (45). See RNA‐seq details in Tables S6–S10. The mean FPKM values between control and stress conditions were statistically compared using Wilcoxon's test. Significance levels were indicated as follows: *p ≤ 0.05, **p ≤ 0.01, ***p ≤ 0.001, and ****p ≤ 0.0001. Only the conditions that have gene expression (fragments per kilobase of transcript per million mapped reads [FPKM] > 0) in over 50% of the samples were included in the statistical test.

To evaluate the expression of the identified ULPs, we examined their transcript levels using data from the plant RNAseq database (Yu et al., 2022: https://plantrnadb.com/ricerna/). We retrieved the FPKM expression values for the identified ULPs (RGAP models) from RNA‐seq data across 631 samples of shoots, leaves, and roots under normal conditions (Table S5). These values were then scaled (*z*‐score) for within‐ULP comparison (Figure 5a), and the *z*‐scores for the three tissues were averaged (Figure 5b). Hierarchical clustering reveals distinct tissue‐specific expression patterns, highlighting the differential regulation of genes in the three rice tissues. Notably, several genes were predominantly expressed in the shoot, while *LOC_Os07g32090 (OsULP43c)*, *LOC_Os04g54670* (*OsULP44a*), *LOC_Os12g36580* (*OsULP40d*), and *LOC_Os09g12480* (*OsULP45a*) exhibited relatively higher expression in the roots (Figure 5a,b). To compare gene expression levels between ULPs, we scaled FPKM values across all the ULPs within the RNA‐seq data. The ULPs that exhibited high expression across all three tissues under normal conditions include *LOC_Os12g01290* (*OsULP36*), *LOC_Os06g29310* (*OsOTS1*), *LOC_Os01g25370* (*OsELS1*), *LOC_Os05g11770* (*OsSPF1*), *LOC_Os03g22400* (*Os_cFUG1*), *LOC_Os11g01180* (*OsULP37a*), *LOC_Os11g10780* (*Os_nFUG1*), and *LOC_Os12g41380* (*OsOTS2*). In contrast, another known ULP, *LOC_Os03g29630* (*OsELS2*), displayed relatively lower expression, but its expression level still remained close to the mean (*z*‐score = 0) (Figure S10a). We next explored two publicly available single‐cell RNA‐seq datasets (Wang et al., 2025; Zhu et al., 2025) to look for evidence of tissue type‐specific expression (Figure S11). Overall, known ULPs (*OsOTS1*, *Os_cFUG1*, *OsELS1*, *OsOTS2*, *OsSPF1*, and *OsELS2*) and two novel ULPs, *OsULP40d* and *OsULP40e* show relatively high transcription levels in almost every cell type (Figure S11a,b). Interestingly, *OsULP36*, *OsULP44c*, *OsULP37a*, and *Os_nFUG1* had some preliminary evidence of tissue‐specific expression (noting this evidence comes from one dataset only), where they had the highest expression in bud tissues (parenchyma cell, primordium, epidermis, and root meristem; Figure S11a‐I). In addition, *Os_nFUG1* show relatively high expression in leaves and young spikes (Figure S11a‐II). *OsULP35* had the highest expression in seeds (plumule, vascular cylinder, and scutellum; Figure S11a‐III). *OsULP50c* was expressed exclusively in leaves and spikelets (Figure S11a‐IV). Focusing on root cells, *Os_nFUG1*, *OsULP40d*, *OsULP50i*, *OsULP35*, and *OsULP36* show relatively higher transcript expression than the other novel ULPs (Figure S11b). Interestingly, *OsULP35* had the highest expression in vascular bundles (phloem, metaxylem, and protoxylem), endodermis, and pericycle (Figure S11b). *Os_nFUG1* had the highest expression detected in the root cap, cortex, and protoxylem, and lowest in trichoblast (Figure S11b). It is thus plausible to hypothesize that newly described ULPs have different functional roles in rice.

OTS1 and OTS2 have been shown to respond to salt and drought stress in both *Arabidopsis* and rice (Srivastava et al., 2017). Additionally, both OTS proteins play roles in bacterial pathogen responses in *Arabidopsis* (Bailey et al., 2016). To determine if other identified rice ULPs also respond to selected abiotic or biotic stresses, we assessed RNA‐seq data from treatments including salt (148 samples), drought (188 samples), heat (54 samples), nematodes (*Meloidogyne graminicola*, *Hirschmanniella*), mycorrhizal fungi and rice stripe virus infection (105 samples), and ABA (45 samples), along with their respective controls (Tables S6–S10; Figure S10).

Overall, the highly expressed ULPs demonstrated responsiveness to the abiotic stresses (Figure 5c). Under salt conditions, we observed significant up‐regulation (Wilcoxon's test, *p*‐value < 0.05) of *LOC_Os01g25370* (*OsELS1*), *LOC_Os06g29310* (*OsOTS1*), *LOC_Os11g10780* (*Os_nFUG1*), and *LOC_Os03g22400* (*Os_cFUG1*). Under drought conditions, *LOC_Os01g25370* (*OsELS1*) and *LOC_Os11g10780* (*Os_nFUG1*) were up‐regulated, similar to their response under salt treatment. In contrast, *LOC_Os06g29310* (*OsOTS1*) was significantly down‐regulated. Heat stress significantly up‐regulated *LOC_Os01g25370* (*OsELS1*), *LOC_Os03g29630 (OsELS2)*, *LOC_Os10g33450 (OsULP35)*, *LOC_Os11g10780* (*Os_nFUG1*), and *LOC_Os12g41380* (*OsOTS2*). On the other hand, heat stress caused the significant down‐regulation of *LOC_Os12g01290 (OsULP36) and LOC_Os06g29310* (*OsOTS1*). Under ABA treatments, a central phytohormone involved in responding to abiotic stresses, we observed significant upregulation of *LOC_Os01g25370* (*OsELS1*), *LOC_Os11g10780* (*Os_nFUG1*), *LOC_Os12g41380* (*OsOTS2*), and *LOC_Os05g11770* (*OsSPF1*). In contrast, *LOC_Os06g29310* (*OsOTS1*) and *LOC_Os11g01180 (OsULP37a)* were down‐regulated.

Under biotic stress conditions, only *OsOTS2* showed significant up‐regulation under the nematode/fungi/virus infection (Figure 5c; Tables S6–S10). In addition, three ULPs (*LOC_Os10g06910*, *LOC_Os09g08440*, and *LOC_Os02g27280*), which are ULP‐domain conserved across RPRP pan‐genome but exhibit low or zero expression levels in the other stresses, showed slightly increased transcription under the pathogen infection. This suggests that these ULPs may be involved in biotic stress responses.

## 4 DISCUSSION

ULPs are one of the key components in SUMOylation process because of their dual functions in maturing SUMO proteins and cleaving SUMOylated proteins (Yates et al., 2016). In *O. sativa*, the identification of ULPs has been previously reported, with some speculation of the ULP expansion during domestication (Garrido et al., 2018; Srivastavaet al., 2016b; Yates et al., 2016). However, previous methods used relatively simple sequence‐based analysis to determine the count of ULP genes/proteins. In this study, we greatly expanded on previous research by taking a multi‐pronged approach, including sequence searching, domain identification, phylogenetics, and structural bioinformatics to validate the presence of the specific protease active site. We explored the ULP gene/protein set within the IRGSP RefSeq and within a population‐aware pan‐genome set (RPRP), demonstrating that the family expansion (compared to model organisms) is consistently observed in multiple varieties. We also observed within the pan‐genome analysis that ULP proteins are commonly found with single or low occupancy (cloud genes) across the pan‐genome, which tends to be indicative of genes under selective breeding pressure. The pan‐gene set analysis of the RPRP genomes also identified particular protein domains as invariable, partially variable, and highly variable across the pan‐genome (based on the mean domain occupancy being < 10 across 16 genomes)—with the highly variable domains characterized by those involved in biotic stress responses (like NB‐ARC and LRR) (Contreras‐Moreira et al., 2025). The ULP protease domain falls into the highly variable domain, as the mean domain occupancy is 5.94, with an enrichment of single‐copy genes (present in only one genome).

In the genus *Oryza*, two rice species have been cultivated: *O. sativa* (Asian rice) and *O. glaberrima* (African rice). The count of ULP genes in Asian cultivated rice is higher than in wild rice. The rice IRGSP RefSeq contains 45 ULPs, and the RPRP genomes (representing the genetic diversity of Asian rice) have ULP counts ranging between 32 and 39. The higher count in the IRGSP RefSeq might be due to the presence of a larger number of genes called from the three sets (RAP‐DB, RGAP, and Gramene), compared to a single gene model set in the RPRP genomes. In contrast to Asian rice, only a single cultivated species exists in Africa (*O. glaberrima*), which shows no evidence of such expansion (11 ULPs). This observation suggests that ULP expansion is a lineage‐specific event in Asian cultivated rice. The similar observation that domestication contributes to gene expansion was also suggested by Jacquemin et al. (2014). The gene counts of seven gene families (NB‐ARC proteins, F‐box proteins, aspartic proteases, BTB/POZ proteins (BTB), trypsin α‐amylase inhibitor proteins (Tryp‐α‐amyl), glutaredoxins, and Zf‐Dof protein) were expanded in *O. sativa* spp. *japonica* and *indica* (AA genome) and *O. glaberrima* (AA genome, cultivated African rice), when compared to *O. brachyantha* (FF genome, wild relative) (Jacquemin et al., 2014).

Wild relatives of rice species have a broader range, with ULP numbers ranging from 9 to 36 ULPs. *O. punctata* is an African wild rice species, considered as a weed (Vaughan et al., 2003), and contains the highest number of ULP (36) among wild rice species. *O. punctata* is classified as BB genome, which is distantly related to RPRP rice (AA genome type). This indicates a divergence of ULP expansion in *Oryza* relatives. Interestingly, *O. punctata* is regarded as having pathogen‐resistant traits and hence has been explored for breeding with cultivated rice varieties (Jena et al., 2016).

The expansion of some ULPs in the Asian rice genome might have been facilitated and diversified by transposable elements (Jiang et al., 2004), as evidenced by the presence of MULE transposase, probable transposase Ptta/En/Spm plant domains, and aminotransferase‐like (plant mobile domain) fused with the ULPs (Figure S7). MULE transposase and probable transposase Ptta/En/Spm plant domains are DNA transposons that use a “cut‐and‐paste” mechanism and might capture a fragment of host genes (e.g., ULP) to reintegrate to a new location, creating gene duplication (Hanada et al., 2009). Ankyrin repeats are protein‐protein interaction motifs that recruit substrates or target proteins (Mosavi et al., 2004). Having this motif fused with ULP domains might mediate substrate targeting and specificity for deSUMOylation.

Asian rice was cultivated about 9000 years ago between two wild rice species: *O. rufipogon* and *O. nivara* (Fornasiero et al., 2022). Over time, beneficial traits such as grain quality, stress, and pathogen resistance were selected through the breeding process (Hernández‐Soto et al., 2021). Genes inherited from these wild ancestors have been identified as domestication‐associated genes (Jing et al., 2023). *Os_cFUG1* is one of the domesticated genes derived from *O. nivara* based on the haplotype network analysis using the single‐nucleotide polymorphisms within the genes and 1 kb upstream and downstream sequences (Jing et al., 2023). However, our phylogenetic analysis of the ULPs suggests that Os_cFUG1 is more closely related to ELS‐type (Figure 2b), and the protein sequence is identical to the *O. rufipogon* orthologue (Figure S6). Based on this observation, we propose a novel *OsFUG1* (*Os11g0214001*, *LOC_Os11g10780*) and *OsELS3* (renaming the current *OsFUG1*; *Os03g0344300*, *LOC_Os03g22400*). In addition, the SUMO‐conjugating enzyme OsSCE1a was identified as a key regulatory protein in 9YJ30 rice, as it has been shown to associate with functional “stay‐green” traits and growth duration (Yuan et al., 2025). These findings highlight the importance of SUMOylation processes and their likely selection during rice domestication.

The public transcriptomic data analysis revealed gene expression patterns of the identified ULP in three main tissues of rice: leaves, shoots, and roots (Figure 5) and different organs/tissues at the single‐cell level (Figure S11). The highly expressed ULPs have domain occupancy generally in at least 15 out of 16 of the pan‐genome set. However, another highly expressed ULP, LOC_*Os12g01290* (*OsULP36*), with eight domain occupancies, is found in Tempjap, Tropjap, Aro, and Aus rice varieties but not in Indica varieties except *Os*Lima. This gene, however, has an unclear gene model, making it uncertain whether any of the source models are accurate (Figure S12). Given the evidence of transcription, further investigation, including gene cloning, protein expression, and SUMO protease assays, will be necessary.

SUMOylation modifies proteins, for example, transcription factors, to regulate their functions (promote or inhibit) during stress responses (Roy & Sadanandom, 2021). For example, SUMOylation of the rice DELLA protein SLR1 at lysine 2 has been found to regulate gene transcriptional levels and increase yield under salt stress (Fernandes et al., 2024). SUMO proteases, on the other hand, work oppositely by deSUMOylating the target proteins (e.g., transcription factors) and resetting the system.

Five ULP genes from our list—Os03g0365250 (LOC_Os03g24960), Os03g0365200 (LOC_Os03g24990), Os04g0583400 (LOC_Os04g49394), Os10g0474000 (LOC_Os10g33450), and Os10g0388600 (LOC_Os10g24954) (Table 1)—were identified as salt‐related candidate genes from comprehensively integrating quantitative trait loci and transcriptomic analyses of salt treatments in *O. sativa* whole seedlings, excluding root samples (Phosuwan et al., 2024), suggesting functions of these proteases under salt stress. However, their transcription levels were low in our list of RNA‐seq datasets (Figure S10b), possibly due to the temporal induction of gene expression. In contrast, the two known salt‐responsive proteins, OsOTS1 (Os06g0487900) and OsOTS2 (Os12g0606900), were not included in their list because these proteins are expressed and responsive to salt exclusively in roots (Srivastava et al., 2017). This highlights the tissue specificity of ULPs in *O. sativa*.

OTS1 and OTS2 act as negative regulators of salicylic acid (SA) signaling in *Arabidopsis* when challenged with the bacterial pathogen *Pseudomonas syringae* (Bailey et al., 2016). During *P. syringae* infection, SA accumulation induces the degradation of OTS1 and OTS2 proteins. Once the infection is managed, the levels of OTS1 and OTS2 proteins are restored to regulate the expression of *ISOCHORISMATE SYNTHASE1* (*ICS1*) to prevent excessive responses to the pathogen (Bailey et al., 2016). We also observed significant upregulation of *OsOTS2* transcripts but non‐significant upregulation of *OsOTS1* in rice infected with nematodes, fungi, and viruses (Figure 5c). This suggests that *OsOTS2* may be upregulated to restore protein levels after overcoming the infection. However, we observed upregulation of other ULPs (Figure 5c), which, although not statistically significant, may still play a role in the pathogen defense mechanism.

In conclusion, our analysis identified 45 ULPs in *O. sativa* (Nipponbare). We also characterized ULPs in wild relatives and RPRP rice at the pan‐genomic level, providing evidence of potential functional ULPs and their transcript expression. Additionally, our study offers insights into the role of these ULPs in response to abiotic and biotic stresses. The differences in gene expression patterns of the novel ULPs (both bulk and single‐cell RNA‐seq) suggest non‐redundant functions and specificities of the protease activities. Experimentally characterizing the deSUMOylation function of these novel ULPs using a SUMO protease assay (Srivastava et al., 2016a) and identifying their target proteins will be crucial for understanding their specificity. Finally, the novel ULPs identified in this study could serve as valuable targets for future rice breeding and other crop improvement.

## AUTHOR CONTRIBUTIONS

**Kawinnat Sue‐ob**: Conceptualization; formal analysis; investigation; methodology; software; visualization; writing—original draft; writing—review and editing. **Eshan Sharma**: Software; visualization; writing—review and editing. **Cunjin Zhang**: Writing—review and editing. **Rahul Bhosale**: Methodology; writing—review and editing. **Ari Sadanandom**: Conceptualization; supervision; writing—review and editing. **Andrew R. Jones**: Conceptualization; methodology; resources; supervision; writing—review and editing.

## CONFLICT OF INTEREST STATEMENT

The authors declare no conflicts of interest.

## Supporting information




**Supplementary Fig. 1** ULP identification workflow.
**Supplementary Fig. 2**. Multiple sequence alignment shows the catalytic sites of well‐characterised ULPs (SUMOylation) that are distinguished from UBPs (Ubiquitination) in *A. thaliana*.
**Supplementary Fig. 3**. 131 Pan‐gene clusters of genes that harbour ULP domains and catalytic sites.
**Supplementary Fig. 4** Multiple sequence alignments of a) OsOTS1 and b) OsELS1 pan‐genes.
**Supplementary Fig. 5** MSA of AtELS1/2, Os_cFUG1, AtFUG1, and Os_nFUG1 (RAP‐DB and Gramene).
**Supplementary Fig. 6**. a) Cluster 26 showing the cluster of Os_cFUG1 b) Os_cFUG1 protein sequence alignment with its orthologues in *O. nivara* and *O. rufipogon*.
**Supplementary Fig. 7**. Only ULP proteins that fused with other protein domains.
**Supplementary Fig. 8**. All ULP protein domains architecture.
**Supplementary Fig. 9**. Genomic location of ULPs in RPRP rice population.
**Supplementary Fig. 10**. Transcriptomic evidence of the novel ULPs in *O. sativa* Japonica Nipponbare.
**Supplementary Fig. 11**. Heatmaps showing expression levels of ULPs in various cell types of rice.
**Supplementary Fig. 12**. Screenshot of *LOC_Os12g01290* gene models derived from (https://panoryza.org/jbrowse/).

**Table S1** Summary information of the RPRP, wild rice and cutgrass genomes used in this study.
**Table S2** Results from BLASTP and TBLASTN of *S.cerevisae* and *A.thaliana* ULPs against *O.sativa* (RAP‐DB and MSU models). The protein sequences contains ULP domain as identified by InterProScan and the catalytic sites are aligned with known ULPs from *S.cerevisiae* and *A. thaliana*.
**Table S3** Order of gene ids as in the phylogenetic tree in figure 2.
**Table S4** The lengths (angstrom, Å) between the three catalytic sites (H, D, and C).
**Table S5** Public transcriptomic data of O.sativa tissues: shoot, leaves, and roots (Yu et al., 2022). The order of the table is the same as the clustered row of the heatmap in Figure 5A.
**Table S6** FPKM of *O.sativa* Japonica RNA‐seq between controls and salt treatments (Yu et al., 2022).
**Table S7** FPKM of O.sativa Japonica RNA‐seq between controls and drought treatments (Yu et al., 2022).
**Table S8** FPKM of O.sativa Japonica RNA‐seq between controls and heat treatments (Yu et al., 2022).
**Table S9** FPKM of O.sativa Japonica RNA‐seq between controls and menatode/fungi/virus infection (Yu et al., 2022).
**Table S10** FPKM of O.sativa Japonica RNA‐seq between controls and ABA treatments (Yu et al., 2022).

## Data Availability

The scripts used in this project are available on GitHub (https://github.com/PGB‐LIV/ULP_identification_rice). The AlphaFold protein models of ULPs from wild relatives of rice and the RPRP rice population are available at https://doi.org/10.5061/dryad.1ns1rn97f.

## References

[tpg270200-bib-0001] Augustine, R. C. , & Vierstra, R. D. (2018). SUMOylation: Re‐wiring the plant nucleus during stress and development. Current Opinion in Plant Biology, 45(Pt A), 143–154. 10.1016/j.pbi.2018.06.006 30014889

[tpg270200-bib-0002] Augustine, R. C. , York, S. L. , Rytz, T. C. , & Vierstra, R. D. (2016). Defining the SUMO system in maize: SUMOylation is up‐regulated during endosperm development and rapidly induced by stress. Plant Physiology, 171(3), 2191–2210. 10.1104/pp.16.00353 27208252 PMC4936565

[tpg270200-bib-0003] Bailey, M. , Srivastava, A. , Conti, L. , Nelis, S. , Zhang, C. , Florance, H. , Love, A. , Milner, J. , Napier, R. , Grant, M. , & Sadanandom, A. (2016). Stability of small ubiquitin‐like modifier (SUMO) proteases OVERLY TOLERANT TO SALT1 and ‐2 modulates salicylic acid signalling and SUMO1/2 conjugation in *Arabidopsis thaliana* . Journal of Experimental Botany, 67(1), 353–363. 10.1093/jxb/erv468 26494731 PMC4682439

[tpg270200-bib-0004] Benlloch, R. , & Lois, L. M. (2018). Sumoylation in plants: Mechanistic insights and its role in drought stress. Journal of Experimental Botany, 69(19), 4539–4554. 10.1093/jxb/ery233 29931319

[tpg270200-bib-0005] Blackburne, B. P. , & Whelan, S. (2013). Class of multiple sequence alignment algorithm affects genomic analysis. Molecular Biology and Evolution, 30(3), 642–653. 10.1093/molbev/mss256 23144040

[tpg270200-bib-0006] Camacho, C. , Coulouris, G. , Avagyan, V. , Ma, N. , Papadopoulos, J. , Bealer, K. , & Madden, T. L. (2009). BLAST+: Architecture and applications. BMC Bioinformatics, 10, 421. 10.1186/1471-2105-10-421 20003500 PMC2803857

[tpg270200-bib-0007] Celen, A. B. , & Sahin, U. (2020). Sumoylation on its 25th anniversary: Mechanisms, pathology, and emerging concepts. FEBS Journal, 287(15), 3110–3140. 10.1111/febs.15319 32255256

[tpg270200-bib-0008] Clark, L. , Sue‐Ob, K. , Mukkawar, V. , Jones, A. R. , & Sadanandom, A. (2022). Understanding SUMO‐mediated adaptive responses in plants to improve crop productivity. Essays in Biochemistry, 66(2), 155–168. 10.1042/EBC20210068 35920279 PMC9400072

[tpg270200-bib-0009] Contreras‐Moreira, B. , Saraf, S. , Naamati, G. , Casas, A. M. , Amberkar, S. S. , Flicek, P. , Jones, A. R. , & Dyer, S. (2023). GET_PANGENES: Calling pangenes from plant genome alignments confirms presence‐absence variation. Genome Biology, 24(1), Article 223. 10.1186/s13059-023-03071-z 37798615 PMC10552430

[tpg270200-bib-0069] Contreras‐Moreira, B. , Sharma, E. , Saraf, S. , Naamati, G. , Gupta, P. , Elser, J. , Chebotarov, D. , Chougule, K. , Lu, Z. , Wei, S. , Olson, A. , Tsang, I. , Lodha, D. , Zhou, Y. , Yu, Z. , Zhao, W. , Zhang, J. , Amberkar, S. , Sue‐Ob, K. , … Jones, A. R. (2025). The PanOryza pangene catalog of Asian cultivated rice. Genome Research, 125. 10.1101/gr.280790.125 PMC12758395 41386984

[tpg270200-bib-0011] Emenecker, R. J. , Griffith, D. , & Holehouse, A. S. (2021). Metapredict: A fast, accurate, and easy‐to‐use predictor of consensus disorder and structure. Biophysical Journal, 120(20), 4312–4319. 10.1016/j.bpj.2021.08.039 34480923 PMC8553642

[tpg270200-bib-0012] Fernandes, T. , Goncalves, N. M. , Matiolli, C. C. , Rodrigues, M. A. A. , Barros, P. M. , Oliveira, M. M. , & Abreu, I. A. (2024). SUMOylation of rice DELLA SLR1 modulates transcriptional responses and improves yield under salt stress. Planta, 260(6), 136. 10.1007/s00425-024-04565-1 39514093 PMC11549141

[tpg270200-bib-0013] Fornasiero, A. , Wing, R. A. , & Ronald, P. (2022). Rice domestication. Current Biology, 32(1), R20–R24. 10.1016/j.cub.2021.11.025 35015986

[tpg270200-bib-0014] Garrido, E. , Srivastava, A. K. , & Sadanandom, A. (2018). Exploiting protein modification systems to boost crop productivity: SUMO proteases in focus. Journal of Experimental Botany, 69(19), 4625–4632. 10.1093/jxb/ery222 29897480 PMC6117578

[tpg270200-bib-0015] Ghimire, S. , Tang, X. , Zhang, N. , Liu, W. , Qi, X. , Fu, X. , & Si, H. (2020). Genomic analysis of the SUMO‐conjugating enzyme and genes under abiotic stress in potato (*Solanum tuberosum* L.). International Journal of Genomics, 2020, 9703638. 10.1155/2020/9703638 32802829 PMC7335410

[tpg270200-bib-0016] Gu, Z. , Eils, R. , & Schlesner, M. (2016). Complex heatmaps reveal patterns and correlations in multidimensional genomic data. Bioinformatics, 32(18), 2847–2849. 10.1093/bioinformatics/btw313 27207943

[tpg270200-bib-0017] Hanada, K. , Vallejo, V. , Nobuta, K. , Slotkin, R. K. , Lisch, D. , Meyers, B. C. , Shiu, S.‐H. , & Jiang, N. (2009). The functional role of pack‐MULEs in rice inferred from purifying selection and expression profile. The Plant Cell, 21(1), 25–38. 10.1105/tpc.108.063206 19136648 PMC2648092

[tpg270200-bib-0018] Hao, Y. , Stuart, T. , Kowalski, M. H. , Choudhary, S. , Hoffman, P. , Hartman, A. , Srivastava, A. , Molla, G. , Madad, S. , & Fernandez‐Granda, C. (2024). Dictionary learning for integrative, multimodal and scalable single‐cell analysis. Nature Biotechnology, 42(2), 293–304. 10.1038/s41587-023-01767-y PMC10928517 37231261

[tpg270200-bib-0019] Hernández‐Soto, A. , Echeverría‐Beirute, F. , Abdelnour‐Esquivel, A. , Valdez‐Melara, M. , Boch, J. , & Gatica‐Arias, A. (2021). Rice breeding in the new era: Comparison of useful agronomic traits. Current Plant Biology, 27, 100211. 10.1016/j.cpb.2021.100211

[tpg270200-bib-0020] Ibrahim, E. I. , Attia, K. A. , Ghazy, A. I. , Itoh, K. , Almajhdi, F. N. , & Al‐Doss, A. A. (2021). Molecular characterization and functional localization of a novel SUMOylation gene in *Oryza sativa* . Biology, 11(1), 53. 10.3390/biology11010053 35053052 PMC8772976

[tpg270200-bib-0021] Ingole, K. D. , Dahale, S. K. , & Bhattacharjee, S. (2021). Proteomic analysis of SUMO1‐SUMOylome changes during defense elicitation in *Arabidopsis* . Journal of Proteomics, 232, 104054. 10.1016/j.jprot.2020.104054 33238213

[tpg270200-bib-0022] Jacquemin, J. , Ammiraju, J. S. , Haberer, G. , Billheimer, D. D. , Yu, Y. , Liu, L. C. , Rivera, L. F. , Mayer, K. , Chen, M. , & Wing, R. A. (2014). Fifteen million years of evolution in the *Oryza* genus shows extensive gene family expansion. Molecular Plant, 7(4), 642–656. 10.1093/mp/sst149 24214894

[tpg270200-bib-0023] Jena, K. K. , Ballesfin, M. L. , & Vinarao, R. B. (2016). Development of *Oryza sativa* L. by *Oryza punctata* Kotschy ex Steud. monosomic addition lines with high value traits by interspecific hybridization. Theoretical and Applied Genetics, 129(10), 1873–1886. 10.1007/s00122-016-2745-8 27318700

[tpg270200-bib-0024] Jiang, N. , Bao, Z. , Zhang, X. , Eddy, S. R. , & Wessler, S. R. (2004). Pack‐MULE transposable elements mediate gene evolution in plants. Nature, 431(7008), 569–573. 10.1038/nature02953 15457261

[tpg270200-bib-0025] Jing, C. Y. , Zhang, F. M. , Wang, X. H. , Wang, M. X. , Zhou, L. , Cai, Z. , Han, J. D. , Geng, M. F. , Yu, W. H. , Jiao, Z. H. , Huang, L. , Liu, R. , Zheng, X. M. , Meng, Q. L. , Ren, N. N. , Zhang, H. X. , Du, Y. S. , Wang, X. , Qiang, C. G. , … Ge, S. (2023). Multiple domestications of Asian rice. Nature Plants, 9(8), 1221–1235. 10.1038/s41477-023-01476-z 37550371

[tpg270200-bib-0026] Jones, P. , Binns, D. , Chang, H. Y. , Fraser, M. , Li, W. , McAnulla, C. , McWilliam, H. , Maslen, J. , Mitchell, A. , Nuka, G. , Pesseat, S. , Quinn, A. F. , Sangrador‐Vegas, A. , Scheremetjew, M. , Yong, S. Y. , Lopez, R. , & Hunter, S. (2014). InterProScan 5: Genome‐scale protein function classification. Bioinformatics, 30(9), 1236–1240. 10.1093/bioinformatics/btu031 24451626 PMC3998142

[tpg270200-bib-0027] Joo, J. , Choi, D. H. , Lee, Y. H. , Seo, H. S. , & Song, S. I. (2019). The rice SUMO conjugating enzymes OsSCE1 and OsSCE3 have opposing effects on drought stress. Journal of Plant Physiology, 240, 152993. 10.1016/j.jplph.2019.152993 31212102

[tpg270200-bib-0066] Kawahara, Y. , de la Bastide, M. , Hamilton, J. P. , Kanamori, H. , McCombie, W. R. , Ouyang, S. , Schwartz, D. C. , Tanaka, T. , Wu, J. , Zhou, S. , Childs, K. L. , Davidson, R. M. , Lin, H. , Quesada‐Ocampo, L. , Vaillancourt, B. , Sakai, H. , Lee, S. S. , Kim, J. , Numa, H. , … Matsumoto, T. (2013). Improvement of the Oryza sativa Nipponbare reference genome using next generation sequence and optical map data. Rice, 6(1), 4. 10.1186/1939-8433-6-4 24280374 PMC5395016

[tpg270200-bib-0028] Kim, D. , Paggi, J. M. , Park, C. , Bennett, C. , & Salzberg, S. L. (2019). Graph‐based genome alignment and genotyping with HISAT2 and HISAT‐genotype. Nature Biotechnology, 37(8), 907–915. 10.1038/s41587-019-0201-4 PMC7605509 31375807

[tpg270200-bib-0029] Kumar, S. , Stecher, G. , Li, M. , Knyaz, C. , & Tamura, K. (2018). MEGA X: Molecular evolutionary genetics analysis across computing platforms. Molecular Biology and Evolution, 35(6), 1547–1549. 10.1093/molbev/msy096 29722887 PMC5967553

[tpg270200-bib-0030] Kurepa, J. , Walker, J. M. , Smalle, J. , Gosink, M. M. , Davis, S. J. , Durham, T. L. , Sung, D. Y. , & Vierstra, R. D. (2003). The small ubiquitin‐like modifier (SUMO) protein modification system in Arabidopsis. Accumulation of SUMO1 and ‐2 conjugates is increased by stress. Journal of Biological Chemistry, 278(9), 6862–6872. 10.1074/jbc.M209694200 12482876

[tpg270200-bib-0031] Letunic, I. , & Bork, P. (2024). Interactive tree of life (iTOL) v6: Recent updates to the phylogenetic tree display and annotation tool. Nucleic Acids Research, 52(W1), W78–W82. 10.1093/nar/gkae268 38613393 PMC11223838

[tpg270200-bib-0032] Li, Y. , Wang, G. , Xu, Z. , Li, J. , Sun, M. , Guo, J. , & Ji, W. (2017). Organization and regulation of soybean SUMOylation system under abiotic stress conditions. Frontiers in Plant Science, 8, 1458. 10.3389/fpls.2017.01458 28878795 PMC5573446

[tpg270200-bib-0033] Lian, W. , Zhan, X. , Chen, D. , Wu, W. , Liu, Q. , Zhang, Y. , Cheng, S. , Lou, X. , Cao, L. , & Hong, Y. (2024). Genome‐wide identification and characterization of the PPPDE gene family in rice. Agronomy, 14(5), 1035. 10.3390/agronomy14051035

[tpg270200-bib-0067] López‐Fernández, H. , Ferreira, P. , Reboiro‐Jato, M. , Vieira, C. P. , & Vieira, J. (2021). The pegi3s Bioinformatics Docker Images Project. In *Practical Applications of Computational Biology & Bioinformatics, 15th International Conference* (PACBB 2021, pp. 31–40). 10.1007/978-3-030-86258-9_4

[tpg270200-bib-0035] Mirdita, M. , Schutze, K. , Moriwaki, Y. , Heo, L. , Ovchinnikov, S. , & Steinegger, M. (2022). ColabFold: Making protein folding accessible to all. Nature Methods, 19(6), 679–682. 10.1038/s41592-022-01488-1 35637307 PMC9184281

[tpg270200-bib-0036] Mishra, N. , Srivastava, A. P. , Esmaeili, N. , Hu, W. , & Shen, G. (2018). Overexpression of the rice gene *OsSIZ1* in Arabidopsis improves drought‐, heat‐, and salt‐tolerance simultaneously. PLoS One, 13(8), e0201716. 10.1371/journal.pone.0201716 30092010 PMC6084956

[tpg270200-bib-0037] Morrell, R. , & Sadanandom, A. (2019). Dealing with stress: A review of plant SUMO proteases. Frontiers in Plant Science, 10, 1122. 10.3389/fpls.2019.01122 31620153 PMC6759571

[tpg270200-bib-0038] Mosavi, L. K. , Cammett, T. J. , Desrosiers, D. C. , & Peng, Z. y. (2004). The ankyrin repeat as molecular architecture for protein recognition. Protein Science, 13(6), 1435–1448. 10.1110/ps.03554604 15152081 PMC2279977

[tpg270200-bib-0039] Nurdiani, D. , Widyajayantie, D. , & Nugroho, S. (2018). OsSCE1 encoding SUMO E2‐conjugating enzyme involves in drought stress response of *Oryza sativa* . Rice Science, 25(2), 73–81. 10.1016/j.rsci.2017.11.002

[tpg270200-bib-0040] Orosa, B. , Yates, G. , Verma, V. , Srivastava, A. K. , Srivastava, M. , Campanaro, A. , De Vega, D. , Fernandes, A. , Zhang, C. , Lee, J. , Bennett, M. J. , & Sadanandom, A. (2018). SUMO conjugation to the pattern recognition receptor FLS2 triggers intracellular signalling in plant innate immunity. Nature Communications, 9(1), 5185. 10.1038/s41467-018-07696-8 PMC6281677 30518761

[tpg270200-bib-0041] Paradis, E. , & Schliep, K. (2019). ape 5.0: An environment for modern phylogenetics and evolutionary analyses in R. Bioinformatics, 35(3), 526–528. 10.1093/bioinformatics/bty633 30016406

[tpg270200-bib-0042] Pei, W. , Jain, A. , Ai, H. , Liu, X. , Feng, B. , Wang, X. , Sun, Y. , Xu, G. , & Sun, S. (2019). *OsSIZ2* regulates nitrogen homeostasis and some of the reproductive traits in rice. Journal of Plant Physiology, 232, 51–60. 10.1016/j.jplph.2018.11.020 30530203

[tpg270200-bib-0043] Pei, W. , Jain, A. , Sun, Y. , Zhang, Z. , Ai, H. , Liu, X. , Wang, H. , Feng, B. , Sun, R. , Zhou, H. , Xu, G. , & Sun, S. (2017). OsSIZ2 exerts regulatory influences on the developmental responses and phosphate homeostasis in rice. Scientific Reports, 7(1), 12280. 10.1038/s41598-017-10274-5 28947784 PMC5612973

[tpg270200-bib-0044] Pertea, M. , Pertea, G. M. , Antonescu, C. M. , Chang, T.‐C. , Mendell, J. T. , & Salzberg, S. L. (2015). StringTie enables improved reconstruction of a transcriptome from RNA‐seq reads. Nature Biotechnology, 33(3), 290–295. 10.1038/nbt.3122 PMC4643835 25690850

[tpg270200-bib-0045] Phosuwan, S. , Nounjan, N. , Theerakulpisut, P. , Siangliw, M. , & Charoensawan, V. (2024). Comparative quantitative trait loci analysis framework reveals relationships between salt stress responsive phenotypes and pathways. Frontiers in Plant Science, 15, 1264909. 10.3389/fpls.2024.1264909 38463565 PMC10920293

[tpg270200-bib-0046] Rosa, M. T. G. , Almeida, D. M. , Pires, I. S. , da Rosa Farias, D. , Martins, A. G. , da Maia, L. C. , de Oliveira, A. C. , Saibo, N. J. M. , Oliveira, M. M. , & Abreu, I. A. (2018). Insights into the transcriptional and post‐transcriptional regulation of the rice SUMOylation machinery and into the role of two rice SUMO proteases. BMC Plant Biology, 18(1), 349. 10.1186/s12870-018-1547-3 30541427 PMC6291987

[tpg270200-bib-0068] Rosa, M. T. , & Abreu, I. A. (2019). Exploring the regulatory levels of SUMOylation to increase crop productivity. Current Opinion in Plant Biology, 49, 43–51. 10.1016/j.pbi.2019.04.009 31177030

[tpg270200-bib-0047] Roy, D. , & Sadanandom, A. (2021). SUMO mediated regulation of transcription factors as a mechanism for transducing environmental cues into cellular signaling in plants. Cellular and Molecular Life Sciences, 78(6), 2641–2664. 10.1007/s00018-020-03723-4 33452901 PMC8004507

[tpg270200-bib-0048] Sakai, H. , Lee, S. S. , Tanaka, T. , Numa, H. , Kim, J. , Kawahara, Y. , Wakimoto, H. , Yang, C. C. , Iwamoto, M. , Abe, T. , Yamada, Y. , Muto, A. , Inokuchi, H. , Ikemura, T. , Matsumoto, T. , Sasaki, T. , & Itoh, T. (2013). Rice annotation project database (RAP‐DB): An integrative and interactive database for rice genomics. Plant & Cell Physiology, 54(2), e6. 10.1093/pcp/pcs183 23299411 PMC3583025

[tpg270200-bib-0049] Schrodinger, L. L. C. (2018). The PyMOL Molecular Graphics Plugin System (Version 2.1.1) [Computer software: https://www.pymol.org]

[tpg270200-bib-0050] Seok, H. Y. , Bae, H. , Kim, T. , Mehdi, S. M. M. , Nguyen, L. V. , Lee, S. Y. , & Moon, Y. H. (2021). Non‐TZF protein AtC3H59/ZFWD3 is involved in seed germination, seedling development, and seed development, interacting with PPPDE family protein Desi1 in Arabidopsis. International Journal of Molecular Sciences, 22(9), 4738. 10.3390/ijms22094738 33947021 PMC8124945

[tpg270200-bib-0070] Shin, E. J. , Shin, H. M. , Nam, E. , Kim, W. S. , Kim, J. , Oh, B. , & Yun, Y. (2012). DeSUMOylating isopeptidase: a second class of SUMO protease. EMBO Reports, 13(4), 339–346. 10.1038/embor.2012.3 22370726 PMC3321169

[tpg270200-bib-0051] Srivastava, A. K. , Zhang, C. , Caine, R. S. , Gray, J. , & Sadanandom, A. (2017). Rice SUMO protease overly tolerant to Salt 1 targets the transcription factor, OsbZIP23 to promote drought tolerance in rice. Plant Journal, 92(6), 1031–1043. 10.1111/tpj.13739 29024118

[tpg270200-bib-0052] Srivastava, A. K. , Zhang, C. , & Sadanandom, A. (2016a). Rice OVERLY TOLERANT TO SALT 1 (OTS1) SUMO protease is a positive regulator of seed germination and root development. Plant Signaling & Behavior, 11(5), e1173301. 10.1080/15592324.2016.1173301 27119209 PMC4973764

[tpg270200-bib-0053] Srivastava, A. K. , Zhang, C. , Yates, G. , Bailey, M. , Brown, A. , & Sadanandom, A. (2016b). SUMO is a critical regulator of salt stress responses in rice. Plant Physiology, 170(4), 2378–2391. 10.1104/pp.15.01530 26869703 PMC4825142

[tpg270200-bib-0054] Sureshkumar, S. , Bandaranayake, C. , Lv, J. , Dent, C. I. , Bhagat, P. K. , Mukherjee, S. , Sarwade, R. , Atri, C. , York, H. M. , Tamizhselvan, P. , Shamaya, N. , Folini, G. , Bergey, B. G. , Yadav, A. S. , Kumar, S. , Grummisch, O. S. , Saini, P. , Yadav, R. K. , Arumugam, S. , … Balasubramanian, S. (2024). SUMO protease FUG1, histone reader AL3 and chromodomain protein LHP1 are integral to repeat expansion‐induced gene silencing in *Arabidopsis thaliana* . Nature Plants, 10(5), 749–759. 10.1038/s41477-024-01672-5 38641663

[tpg270200-bib-0055] Teramura, H. , Yamada, K. , Ito, K. , Kasahara, K. , Kikuchi, T. , Kioka, N. , Fukuda, M. , Kusano, H. , Tanaka, K. , & Shimada, H. (2021). Characterization of novel SUMO family genes in the rice genome. Genes & Genetic Systems, 96(1), 25–32. 10.1266/ggs.20-00034 33731501

[tpg270200-bib-0056] Vaughan, D. A. , Morishima, H. , & Kadowaki, K. (2003). Diversity in the *Oryza* genus. Current Opinion in Plant Biology, 6(2), 139–146. 10.1016/S1369-5266(03)00009-8 12667870

[tpg270200-bib-0057] Verma, V. , Croley, F. , & Sadanandom, A. (2018). Fifty shades of SUMO: Its role in immunity and at the fulcrum of the growth–defence balance. Molecular Plant Pathology, 19(6), 1537–1544. 10.1111/mpp.12625 29024335 PMC6637990

[tpg270200-bib-0058] Wang, H. , Sun, R. , Cao, Y. , Pei, W. , Sun, Y. , Zhou, H. , Wu, X. , Zhang, F. , Luo, L. , Shen, Q. , Xu, G. , & Sun, S. (2015). *OsSIZ1*, a SUMO E3 Ligase Gene, is Involved in the Regulation of the Responses to Phosphate and Nitrogen in Rice. Plant & Cell Physiology, 56(12), 2381–2395. 10.1093/pcp/pcv162 26615033

[tpg270200-bib-0059] Wang, X. , Huang, H. , Jiang, S. , Kang, J. , Li, D. , Wang, K. , Xie, S. , Tong, C. , Liu, C. , Hu, G. , Li, H. , Li, C. , Yang, L. , Ding, Y. , Li, S. T. , Wang, F. , Lohmann, J. U. , Liang, Z. , & Gu, X. (2025). A single‐cell multi‐omics atlas of rice. Nature, 644(8077), 722–730. 10.1038/s41586-025-09251-0 40634611

[tpg270200-bib-0060] Yates, G. , Srivastava, A. K. , & Sadanandom, A. (2016). SUMO proteases: Uncovering the roles of deSUMOylation in plants. Journal of Experimental Botany, 67(9), 2541–2548. 10.1093/jxb/erw092 27012284

[tpg270200-bib-0061] Yu, Y. , Zhang, H. , Long, Y. , Shu, Y. , & Zhai, J. (2022). Plant public RNA‐seq database: A comprehensive online database for expression analysis of ∼45 000 plant public RNA‐Seq libraries. Plant Biotechnology Journal, 20(5), 806–808. 10.1111/pbi.13798 35218297 PMC9055819

[tpg270200-bib-0062] Yuan, X. , Luan, Y. , Liu, D. , Wang, J. , Peng, J. , Zhao, J. , Li, L. , Su, J. , Xiao, Y. , Li, Y. , Ma, X. , Zhu, X. , Tan, L. , Liu, F. , Sun, H. , Gu, P. , Xu, R. , Zhang, P. , Zhu, Z. , … Zhang, K. (2025). The SUMO‐conjugating enzyme OsSCE1a from wild rice regulates the functional stay‐green trait in rice. Plant Biotechnology Journal, 23(2), 615–631. 10.1111/pbi.14524 39585184 PMC11772321

[tpg270200-bib-0063] Zhang, S. , Wang, S. , Lv, J. , Liu, Z. , Wang, Y. , Ma, N. , & Meng, Q. (2018). SUMO E3 Ligase SlSIZ1 facilitates heat tolerance in tomato. Plant & Cell Physiology, 59(1), 58–71. 10.1093/pcp/pcx160 29069432

[tpg270200-bib-0064] Zhou, Y. , Yu, Z. , Chebotarov, D. , Chougule, K. , Lu, Z. , Rivera, L. F. , Kathiresan, N. , Al‐Bader, N. , Mohammed, N. , Alsantely, A. , Mussurova, S. , Santos, J. , Thimma, M. , Troukhan, M. , Fornasiero, A. , Green, C. D. , Copetti, D. , Kudrna, D. , Llaca, V. , … Wing, R. A. (2023). Pan‐genome inversion index reveals evolutionary insights into the subpopulation structure of Asian rice. Nature Communications, 14(1), 1567. 10.1038/s41467-023-37004-y PMC10030860 36944612

[tpg270200-bib-0065] Zhu, M. , Hsu, C.‐W. , Peralta Ogorek, L. L. , Taylor, I. W. , La Cavera, S. , Oliveira, D. M. , Verma, L. , Mehra, P. , Mijar, M. , Sadanandom, A. , Perez‐Cota, F. , Boerjan, W. , Nolan, T. M. , Bennett, M. J. , Benfey, P. N. , & Pandey, B. K. (2025). Single‐cell transcriptomics reveal how root tissues adapt to soil stress. Nature, 642(8068), 721–729. 10.1038/s41586-025-08941-z 40307555 PMC12176638

