# Supplementary material for: Ubiquitin‐like SUMO protease expansion in rice (Oryza sativa L.)
Source: Plant Genome. 2026 Feb 12;19(1):e70200. doi: 10.1002/tpg2.70200 (PMC12903978; doi:10.1002/tpg2.70200)
Supplement: Supplementary file 1 [file TPG2-19-e70200-s002.docx]

**Supplementary Figures**

**Supplementary Fig. 1 ULP identification workflow** Identification of ULPs by sequence-based and validation by structure-based searches in 2 rice reference genomes. Known ULP sequences from yeast and *Arabidopsis* were queried to BLASTP and TBLASTN searches in two *O. sativa* reference genomes (RAP-DB and RGAP). The significant sequence hits (e-value < 0.05) from BLAST results were scanned by InterProScan and only the sequences with ULP domains were retained. The ULP domain containing proteins were also obtained from the annotations RAP-DB and RGAP. The ULP candidates from sequence-based searches were aligned to known ULPs from yeast and *Arabidopsis* using MUSCLE, and only the sequences with the right catalytic sites were carried on. We then searched for the ULPs orthologs in RPRP genomes and wild rice populations, using similar steps and cut offs; BLASTP and InterProScan. The proteins with ULPs catalytic domains were aligned with known ULPs from yeast and *Arabidopsis*. Lastly, the representative ULPs from pan-gene clusters and all identified ones in wild rice had protein 3D structures predicted, and the ones with correct catalytic areas were retained.

**Supplementary Fig. 2.** Multiple sequence alignment shows the catalytic sites of well-characterised ULPs (SUMOylation) that are distinguished from UBPs (Ubiquitination) in *A. thaliana*. The protein sequences of UBPs and ULPs were aligned (MUSCLE) and visualised in Jalview. The catalytic sites of ULPs and UBPs are highlighted in red. ULPs have HxDxC while UBPs have CxQxLxH forming catalytic sites (Zhou et al., 2017).

**Supplementary Fig. 3.** 131 Pan-gene clusters of genes that harbour ULP domains and catalytic sites. The individual clusters were aligned by using three MSA tools (MUSCLE, MAFFT, and ClustalOmega) with known ULPs of yeast and *Arabidopsis*. The total number of genes that have correct catalytic sites are also illustrated by the gradient of blues. The pan-gene clusters are separated by the “genome occupancy” within the RPRP populations. The pan-gene clusters for which their protein structure (AlphaFold) suggests wrong catalytic sites are underlined.

**Supplementary Fig. 4** Multiple sequence alignments of a) OsOTS1 and b) OsELS1 pan-genes. The protein sequences were aligned using MUSCLE v5 and visualised in Jalview. The catalytic sites are indicated by red boxes.

**Supplementary Fig. 5** MSA of AtELS1/2, Os_cFUG1, AtFUG1, and Os_nFUG1 (RAP-DB and Gramene). The protein sequences were aligned using MUSCLE v5 and visualised in Jalview.

**Supplementary Fig. 6.** a) Cluster 26 showing the cluster of Os_cFUG1 b) Os_cFUG1 protein sequence alignment with its orthologues in *O. nivara* and *O. rufipogon*. The protein sequences were aligned with MUSCLE, and two mismatches were highlighted in red boxes.

**Supplementary Fig. 7**. Only ULP proteins that fused with other protein domains. The representative ULPs from pan-Oryza (RPRP) and wild rice relatives, which contain other protein domains than ULP as identified by InterProScan, are shown. Colour strips represent species (*S. cerevisiae*, *A. thaliana*, *L. perrieri*, wild rice, African rice, Temperate Japonica (Temjap), Tropical Japonica (Tropjap), Aro rice, Aus rice and Indica rice) and clusters according to the gene tree in Fig. 2b.

**Supplementary Fig. 8.** All ULP protein domains architecture. a) Disorder regions prediction by metapredict. Scores are ranging from 0 to 1 (light blue) which indicates disorder regions. b) Protein domains identified by InterProScan. The ULP proteins are ordered by the gene tree in Fig. 3 .c) The length (amino acids) of ULPs. d) The length of ULP domains (amino acids). E) the ULP domains position on the ULP proteins. Colours represent species; *S. cerevisiae*, *A. thaliana, L. perrieri,* wild rice, African rice, Temperate Japonica (Temjap), Tropical Japonica (Tropjap), Aro rice, Aus rice and Indica rice.

**Supplementary Fig. 9.** Genomic location of ULPs in RPRP rice population. *O. sativa* RPRP genome set: Aro (OsARC), Indica (OsGoSa, OsIR64, OsKYG, OsLaMu, OsLima, OsLiXu, OsMH63, OsPr106, and OsZS97), Aus (OsN22, OsNaBo), Tropical Japonica or Tropjap (OsAzu, OsCMeo, OsKeNa), Temperate Japonica or Tempjap (ISRGP RefSeq), see genome details in Table S1.

**Supplementary Fig. 10.** Transcriptomic evidence of the novel ULPs in *O. sativa* Japonica Nipponbare. a) A total 631 publicly available RNA-seq datasets of *O. sativa* leaves (315), shoots (116), and roots (200) showing tissue-specific patterns of the novel ULPs. The FPKM values were scaled by RNA-seq (z-score by row) then clustered by tissues (row) and within the domain occupancy groups (1-16). b) The FPKM values of all ULPs under control (blue) and 5 stress treatments (red): salt (148), drought (188), heat (54), ABA (45), and nematode/fungi/virus infection (105). See RNA-seq details in **Table S6-10**. The mean FPKM values between control and stress conditions were statistically compared using Wilcoxon’s test. Significance levels were indicated as follows: p ≤ 0.05 (*), p ≤0.01 (**), p ≤ 0.001 (***), and p ≤ 0.0001 (****). Only the conditions that have gene expression (FPKM > 0) in over 50% of the samples were included in the statistical test.

**Supplementary Fig. 11.** Heatmaps showing expression levels of ULPs in various cell types of rice. The heatmaps show log-normalised expression values for each mentioned cell type from wild type a) different rice organs (GSE232863) and b) rice roots (GSE251706). The rows and columns were clustered based on Euclidean distance. SAM; shoot apical meristem, SP;	young spike, ST; Spikelet.

**Supplementary Fig. 12.** Screenshot of *LOC_Os12g01290* gene models derived from (<https://panoryza.org/jbrowse/>). The transcriptomic evidence is shown in pink and blue (pair-ended RNAseq).
